# A conditional mutant of the fatty acid synthase unveils unexpected cross talks in mycobacterial lipid metabolism

**DOI:** 10.1098/rsob.160277

**Published:** 2017-02-22

**Authors:** Matías Cabruja, Sonia Mondino, Yi Ting Tsai, Julia Lara, Hugo Gramajo, Gabriela Gago

**Affiliations:** Laboratory of Physiology and Genetics of Actinomycetes, Instituto de Biología Molecular y Celular de Rosario (IBR-CONICET), Facultad de Ciencias Bioquímicas y Farmacéuticas, Universidad Nacional de Rosario, Rosario, Argentina

**Keywords:** fatty acid synthase I, tuberculosis, mycolic acids

## Abstract

Unlike most bacteria, mycobacteria rely on the multi-domain enzyme eukaryote-like fatty acid synthase I (FAS I) to make fatty acids de novo. These metabolites are precursors of the biosynthesis of most of the lipids present both in the complex mycobacteria cell wall and in the storage lipids inside the cell. In order to study the role of the type I FAS system in *Mycobacterium* lipid metabolism *in vivo*, we constructed a conditional mutant in the *fas-acpS* operon of *Mycobacterium smegmatis* and analysed in detail the impact of reduced de novo fatty acid biosynthesis on the global architecture of the cell envelope. As expected, the mutant exhibited growth defect in the non-permissive condition that correlated well with the lower expression of *fas-acpS* and the concomitant reduction of FAS I, confirming that FAS I is essential for survival. The reduction observed in FAS I provoked an accumulation of its substrates, acetyl-CoA and malonyl-CoA, and a strong reduction of C_12_ to C_18_ acyl-CoAs, but not of long-chain acyl-CoAs (C_19_ to C_24_). The most intriguing result was the ability of the mutant to keep synthesizing mycolic acids when fatty acid biosynthesis was impaired. A detailed comparative lipidomic analysis showed that although reduced FAS I levels had a strong impact on fatty acid and phospholipid biosynthesis, mycolic acids were still being synthesized in the mutant, although with a different relative species distribution. However, when triacylglycerol degradation was inhibited, mycolic acid biosynthesis was significantly reduced, suggesting that storage lipids could be an intracellular reservoir of fatty acids for the biosynthesis of complex lipids in mycobacteria. Understanding the interaction between FAS I and the metabolic pathways that rely on FAS I products is a key step to better understand how lipid homeostasis is regulated in this microorganism and how this regulation could play a role during infection in pathogenic mycobacteria.

## 1. Background

All organisms that produce fatty acids (FA) do so via a repeated cycle of reactions. The first committed step in FA biosynthesis is the carboxylation of acetyl-CoA by acetyl-CoA carboxylase (ACC), to form the common extender unit malonyl-CoA. This building block is subsequently condensed and reduced in an iterative fashion until the FA chain matures for use by the cell. In eukaryotes, these reactions are catalysed by a type I fatty acid synthase (FAS I), a large multifunctional protein to which the growing chain is covalently attached [1]. By contrast, most bacteria contain a type II system (FAS II) in which each reaction is catalysed by a discrete protein [2]. Remarkable exceptions to this rule are members of the *Corynebacterineae*, which possess a eukaryotic-like type I FAS [3]. In mycobacteria, FA biosynthesis is initiated by the multifunctional FAS I enzyme, which catalyses the de novo synthesis of medium and long-chain acyl-CoAs from acetyl-CoA, using malonyl-CoA as extender unit. These acyl-CoAs are subsequently used for the synthesis of membrane phospholipids (PL) or they are shuffled into the FAS II system for the synthesis of mycolic acids. In mycobacteria, the type II FAS system is responsible for the elongation of these acyl-CoAs through iterative condensation reactions with malonyl-ACP, leading to the synthesis of the very long-chain meromycolyl-ACPs [4–6]. A remarkable characteristic of the mycobacterial FAS I system is the bimodal behaviour that was demonstrated *in vitro*. Besides producing medium chain acyl-CoAs, the FAS I system releases C_24:0_-CoA in *Mycobacterium smegmatis* [7,8] or C_26:0_-CoA in *Mycobacterium tuberculosis* [9] that becomes carboxylated by a dedicated long chain acyl-CoA carboxylase (LCC) complex to yield an α-carboxy-C_24/26_-CoA. These carboxy-acyl-CoAs are then condensated with the meromycolyl-AMP in a reaction catalysed by the polyketide synthase Pks13 (electronic supplementary material, figure S1), to produce mycolic acids (MA) and their glyco-derivatives, lipids of extreme importance for the maintenance of *M. tuberculosis* membrane properties and immunopathogenicity [10].

To add a higher level of complexity to *M. tuberculosis* lipid metabolism, acyl-CoAs synthesized by FAS I are not only incorporated into PL and/or into the storage lipids triacylglycerides (TAG), but also used as important biosynthetic precursors to produce virulence associated polyketide lipids like phtiocerol-dimycoseroic acid (PDIM), poly-acylated trehalose (PATS) and sulfolipids (SL) [10,11] (electronic supplementary material, figure S1).

The necessary crosstalk between the FAS I and FAS II systems, the different polyketide synthases (PKS) and the TAG biosynthesis pathways expands the complexity of the putative regulatory network that maintain lipid homeostasis in *M. tuberculosis.* It also highlights the relevance of studying the different components and molecular mechanisms that control the homeostasis of lipid metabolism in order to better understand one of the key metabolisms of this pathogen and its relationship with its virulence. Only fragmented information is currently available about the mechanisms involved in the regulation of FA and MA metabolism in response to environmental conditions. Until now, several pieces of evidence have suggested that post-translational modifications using a Ser/Thr protein kinase (STPK)-mediated phosphorylation conduct a tight regulation of MA biosynthetic enzyme activity [12–15]. Additionally, a few transcriptional regulators of MA metabolism have been identified, such as FasR that controls *fas* expression [16], and FadR and MabR that are regulators of the *fasII* operon expression [17,18]. The fact that both MabR and FasR are essential for *M. smegmatis* viability indicates that keeping a tight balance between the activities of the two FAS systems is crucial for mycobacteria metabolism.

In order to study the role of the de novo FA biosynthesis (i.e. the role of the type I FAS system in *Mycobacterium* lipid metabolism *in vivo*) we constructed a conditional mutant in the *fas* gene and analysed in detail the impact of reduced de novo FA biosynthesis on MA biosynthesis and on the global architecture of the cell envelope. Our results demonstrate the essentiality of FAS I for *Mycobacterium* viability and the relevance of this system as a central player in the maintenance of the correct balance between the different mycobacterial lipids.

## 2. Material and methods

### 2.1. Bacterial strains, culture and transformation conditions

The *E. coli* strain DH5α [19] was used for routine sub-cloning and was transformed according to Sambrook *et al.* [20]. *Mycobacterium smegmatis* mc^2^155 is an electroporation-efficient mutant of mc26 [21]. Liquid cultures of *M. smegmatis* mc^2^155, WT-pFRA42B and *fas* cKD were grown at 37°C in 7H9 medium supplemented with 0.2% glycerol and 0.03% Tyloxapol. Hygromycin (50 µg ml^−1^), apramycin (Am, 50 µg ml^−1^), kanamycin (15 µg ml^−1^), streptomycin (Sm, 12 µg ml^−1^) or anhydrotetracycline (ATc, 50–200 ng ml^−1^) were added when needed. Recombinant plasmids and strains genotypes are listed in electronic supplementary material, tables S1 and S2.

### 2.2. DNA manipulation, plasmid construction and mutant generation

Isolation of plasmid DNA, restriction enzyme digestion and agarose gel electrophoresis were carried out by conventional methods [20]. Genomic DNA of *M. smegmatis* was obtained as described previously [22].

For the construction of the *M. smegmatis* mutant allele P*tr*:*fas*_MS_, the 5′ region of the *M. smegmatis fas* gene (*Msmeg_4757*) was amplified with the primers FasMS-FNsiI (5′-TGGATGCATGTGACGATCTACGAACACGA-3′) and FasMS-RXbaI (5′-GGTCTAGACTGCAGCTTCCACAGGTAGG-3′). The 1517 bp PCR product was cloned in pCR BluntII TOPO (Invitrogen) and digested with *Nsi*I/*Xba*I. The fragment was inserted in a pMP349 derivative plasmid that contains P*tr* from pFRA50 cloned into *EcoR*I/*Pvu*II sites, obtaining pFR42 plasmid. This plasmid has been sequenced in order to see that no errors were introduced during amplification. Finally, pFR42 was digested with *Spe*I/*Xba*I and cloned in the *ts* plasmid pPR27 [23], generating pFR18 plasmid. This plasmid was used to transform *M. smegmatis* cells harbouring the plasmid pFRA42B carrying the TetR/Pip OFF system (WT-pFRA42B) [24]. One of the Sm/Am-resistant transformants was grown at 30°C and plated at 42°C to promote plasmid recombination. The recombination event that left *fas* under the control of ATc was confirmed by PCR and the resultant strain was named *fas* cKD.

### 2.3. Determination of FAS I and FAS II activities using *in vitro* radiometric studies of [2-^14^C] malonyl-CoA incorporation

FAS I and FAS II activities were monitored by measuring [2-^14^C] malonyl-CoA incorporation into fatty acid products following the methods of Bloch [25] and Wheeler *et al.* [26]. [2-^14^C] Malonyl-CoA (0.37 MBq ml^−1^) was used and all activities were calculated as pmol of malonyl-CoA incorporated into fatty acid (mg protein)^−1^. Cell-free extracts were prepared from *fas* cKD mutant strain grown in the absence and presence of ATc 200 ng ml^−1^ at T3 and T4. Cells harvested by centrifugation were resuspended in extraction buffer (100 mM potassium phosphate buffer, pH 7.5, 5 mM DTT, 1 mM EDTA, 5 mM MgCl_2_) and disrupted by sonication. The extract was clarified by centrifugation at 25 000*g* for 30 min followed by ultracentrifugation at 40 000*g* for 1 h. The soluble fraction was washed three times with extraction buffer by filtration through an Ultra-Centrifugal Filter Unit (Amicon; Ultra-15; molecular weight cut off 10 000 Da). Protein content was determined using Quant-iT Protein Assay Kits and Qubit fluorometer (Invitrogen) and the results were normalized by OD_600_.

FAS I assay solution: 1 mg of protein extract, 300 µM acetyl-CoA, 20 µM malonyl-CoA, 5 mM DTT, 5 mM EDTA, 1 µM flavin mononucleotide, 0.5 mM β-cyclodextrin, 100 µM NADPH, 100 µM NADH, 50 000 cpm [2-^14^C] malonyl-CoA (Perkin Elmer) and 100 mM potassium phosphate buffer, pH 7, in a total volume of 500 µl.

FAS II assay solution: 1 mg of protein extract, 40 µM malonyl-CoA, 100 µM palmitoyl-CoA, 5 mM DTT, 5 mM EDTA, 140 µM NADPH, 140 µM NADH, 10 µM Coenzyme A (Sigma), 90 µg AcpM, 100 000 cpm [2-^14^C] malonyl-CoA and 100 mM potassium phosphate buffer, pH 7, in a total volume of 500 µl.

Fatty acid synthesis was allowed to proceed for 30 min at 37°C. Fatty acids were separated following saponification (0.5 ml 20% KOH-50% methanol, 30 min, 100°C), acidification (0.3 ml 6 M HCl) and extraction (petroleum ether, four times with 1 ml each time). The extracted fatty acids were transferred to scintillation vials, dried at 50°C and dissolved in 0.2 ml of diethyl ether. Radioactivity was measured by scintillation counting after the addition of 1 ml of scintillation liquid (Perkin Elmer).

### 2.4. RNA techniques

RNA was extracted from *M. smegmatis fas* cKD and WT-pFRA42B grown in the presence and absence of 200 ng ml^−1^ ATc using Direct Zol RNA MiniPrep (Zymo Research).

qRT-PCR was performed using second strand cDNA as template, generated with SuperScript III Reverse Transcriptase (Invitrogen), random primers and a green fluorochrome as the indicator dye (qPCR master mix, Biodynamics). The expression of *fas, acpS, fasR, inhA, fabH, hadB, fabD*, *acpM*, *kasA* and *kasB* was quantified after normalization of RNA levels to the expression of *sigA* gene as previously described [27]. qPCR cycling conditions were as follows: 95°C for 2 min followed by 40 cycles of 95°C for 15 s, 58°C for 15 s and 68°C for 20 s. qPCR data are presented as fold difference of expression in *fas* cKD grown with ATc over that in the same strain grown without ATc 200 ng ml^−1^, using the Pfaffl method [28]. *sigA* was the reference gene used for normalization of the expression level of each target gene. The sequences of all the primers used are listed in electronic supplementary material, table S3.

### 2.5. Lipid analysis

#### 2.5.1. Fatty acid and mycolic acid analysis using thin layer chromatography

Fatty acid and mycolic acid biosynthesis were analysed by incorporation of [1-^14^C] acetate. *Mycobacterium smegmatis fas* cKD and WT-pFRA42B were grown to OD_600_ 0.2 and each culture was split into two equal fractions. ATc 200 ng ml^−1^ was added to one of the cultures while the other served as the untreated control. All of the cultures were incubated at 42°C with gentle shaking (180 r.p.m.). At specific time points of the growth curves, aliquots of 5 ml were radiolabelled with 1 µCi ml^−1^ of [1-^14^C] acetate (50.5 mCi mmol^−1^; Pelkin Elmer) for 1 h at 42°C. Cells were then harvested by centrifugation, washed with phosphate buffer 0.1 M pH 7.6 and stored at −80°C. Fatty acid methyl esters (FAMEs) and mycolic acid methyl esters (MAMEs) were obtained after treatment of the radiolabelled cell pellets containing the same number of cells, with aqueous tetrabutyl ammonium hydroxide followed by esterification with methyl iodide and extraction with dichloromethane, as previously described [29]. The resulting solution of FAMEs and MAMEs was assayed for radioactivity in a Beckman liquid scintillation counter and then subjected to thin layer chromatography (TLC), using silica gel plates (TLC silica gel 60 F_254_, Merck) and hexane:ethyl acetate (9 : 1, v/v) as the developing solvent. Autoradiograms were produced by overnight exposure of the TLC plates to Carestream Kodak BioMax MR films. The developed films were then digitalized and the spots of ^14^C-labelled FAMEs and MAMEs were quantified using Gel-Pro Analyzer software.

### 2.6. Triacylglycerol analysis

The biosynthesis of triacylglycerols (TAGs) was analysed by incorporation of [1-^14^C] acetate. *Mycobacterium smegmatis fas* cKD and WT-pFRA42B were grown to OD_600_ ∼ 0.2 and each culture was split into two equal fractions. ATc 200 ng ml^−1^ was added to one of the fractions while the other served as the untreated control. All of the cultures were incubated at 42°C with gentle shaking (180 r.p.m.). At different time points during the growth of the cultures, aliquots of 5 ml were radiolabelled with 1 µCi ml^−1^ of [1-^14^C] acetate (50.5 mCi mmol^−1^; Perkin Elmer) for 1 h at 42°C. Cells were then harvested by centrifugation, washed with phosphate buffer 0.1 M pH 7.6 and stored at −80°C. Total lipids were extracted from radiolabelled cell pellets containing the same number of cells, with methanol/chloroform (2 : 1 v/v) as described by Bligh & Dyer [30]. After extraction, the lipids were dried and analysed by TLC on silica gel 60 F_254_ plates (Merck), using hexane/diethylether/acetic acid (75 : 25 : 1, v/v/v) as the developing solvent. Autoradiograms were produced by overnight exposure of the TLC plates to Carestream Kodak BioMax MR films.

### 2.7. Fatty acid analysis

To analyse the fatty acid content, the FAMEs were extracted as previously reported [29] and quantified by GC-MS in a Shimadzu GCMS-QP2010 Plus chromatography-mass spectrometer on a SPB-1 capillary column (28 m × 0.25 mm inside diameter). Helium at 1 ml min^−1^ was used as the carrier gas and the column was programmed at 6°C min^−1^ from 40°C to 310°C.

### 2.8. Acyl-CoA analysis

Acyl-CoAs were extracted following the protocol described by Cabruja *et al.* [31]. Bacterial cultures were harvested by centrifugation at 5800*g* for 15 min at room temperature. 0.625 nmol of ^13^C_2:0_-CoA and ^13^C_16:0_-CoA were added to each sample as internal standards. Separation of the different chain-length acyl CoAs was made by ion pairing-reverse phase high performance liquid chromatography (IP-RP-HPLC) with an Agilent 1200 SL instrument (Agilent Corporation, Santa Clara, CA, USA) having a Hypersil GOLD C_18_ column (dimensions: 2.1 mm × 150 mm × 3 µm, Thermo Fisher Scientific, Waltham, MA, USA) at 30°C. High-resolution mass spectrometry was performed in a Bruker micrOTOF-QII, a Q-TOF instrument with an electrospray ionization source (ESI). ESI parameters were optimized for acyl CoA's detection as follows: nebulizer pressure 1.0 bar, desolvation gas (N_2_) flow 4.0 l min^−1^, dry heater 200°C, capillary voltage 2800 V, end plate offset −500 V. The target mass scan was set from 50 to 3000 m z^−1^. The mass spectrometer parameters were set to detect the entire acyl CoA's ions in the single deprotonated form (M−H)− operating in the negative mode.

### 2.9. Total lipid analysis

Total lipid extractions for lipidomic analysis were prepared as follows: cell pellets were washed with 50 mM ammonium acetate, pH 7.8 and transferred to 10 ml glass tubes containing 5 ml CHCl_3_/CH_3_OH (2 : 1, v/v). The samples were incubated overnight at 4°C with gentle agitation. After centrifugation, bacterial pellets were subjected to an additional extraction using CHCl_3_ : CH_3_OH (1 : 2, v/v) for 2 h. Organic extracts were pooled and dried under nitrogen at 4°C. Lipids were resuspended in 3 ml of CHCl_3_, washed with 3 ml of H_2_O and the organic phase was transferred to pre-weighted glass tubes, dried under nitrogen at 4°C and reweighted on a microbalance. Extracts were dissolved in CHCl_3_:CH_3_OH (1 : 1, v/v) at 1 mg ml^−1^ and centrifuged at 3000*g* for 5 min. Lipids were analysed in an Agilent 1200 series HPLC system with a Reprospher (Dr Maish) 100 C8 column (1.8 µm × 50 mm × 2 mm). The flow rate was 0.3 ml min^−1^ in binary gradient mode with the following elution programme: the column was equilibrated with 100% mobile phase A [CH_3_OH:H_2_O (99 : 1, v/v), containing 0.05 mM AcNH_4_]. 2 µl of each sample, containing 13 µg of total lipids in CHCl_3_, were injected, and the same elution conditions continued for 1 min, followed by a 12 min gradient to 100% mobile phase B (isopropanol:hexane:H_2_O (79 : 20 : 1, v/v), containing 0.05 mM AcNH_4_) and holding 1 min at that condition. The column was equilibrated for 2 min with 100% mobile phase A before injection of the next sample. An Agilent 6500 series Q-TOF instrument with a Dual AJS ESI was used for mass analysis. Ionization gas temperature was maintained at 200°C with a 14 l min^−1^ drying gas flow, a 35 psig nebulizer pressure and 3500 volts. Spectra were collected in positive and negative mode from *m*/*z* 115 to 3000 at 4 spectra s^−1^. Continuous infusion calibrants included *m*/*z* 121.051 and 922.010 in positive-ion mode and *m*/*z* 119.035 and 955.972 in negative-ion mode. CID-MS was carried out with an energy of 50 volts. For the comparative analysis, the column is conditioned by four successive mock injections with solvent cycling before randomized QCs and mycobacterial samples are analysed.

### 2.10. Statistical analysis

Data are reported as arithmetic means of the results obtained from three independent experiments ± standard deviations. Statistical significance was calculated using *t*-test or ANOVA analysis as described in the figure legends. Statistical significance was accepted at *p* < 0.05.

## 3. Results

### 3.1. Construction and growth behaviour of a *fas* conditional mutant in *Mycobacterium smegmatis*

**Figure 1.**
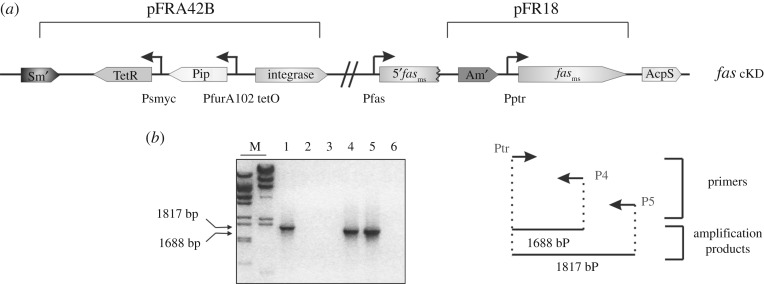
(*a*) Schematic representation of *Mycobacterium smegmatis* conditional mutant *fas* cKD. The 5′ end of *fas* was cloned under the control of the P*tr* in the *ts* plasmid pPR27, obtaining the plasmid pFR18. This plasmid was used to transform *M. smegmatis* cells harbouring the plasmid pFRA42B carrying the TetR/Pip OFF system. (*b*) The correct genetic organization of the *M. smegmatis* conditional mutant *fas* cKD was verified by PCR. The amplification was performed using a genomic DNA of a representative Sm^r^ Am^r^ clone, named *fas* cKD, and primers specific for the P*tr* promoter (Ptr) and the *fas* gene (P4 and P5). Primer P5 hybridizes to a region of the *fas* gene not present in the plasmid used for the allelic exchange experiment. In these reactions, amplification products appear only if the expected allelic exchange occurred. By contrast, no amplification products are expected in the case of an ectopic recombination. PCR control reactions were performed using primers Ptr and P4 or P5 with the plasmid pFR18 as a template. Lanes 1 and 4: amplification of genomic DNA of *M. smegmatis fas* cKD using primers Ptr and P5 (lane 1) or Ptr and P4 (lane 4). Lanes 2 and 5: amplification of plasmid pFR18 using primers Ptr and P5 (lane 2) or Ptr and P4 (lane 5). Lanes 3 and 6: negative controls.

In order to study the effect of impairing the de novo FA biosynthesis on *M. smegmatis* lipid homeostasis, we constructed an *M. smegmatis* conditional knockdown mutant of the *fas-acpS* operon. For this, the *fas* promoter was replaced by the repressible promoter P*ptr* in a strain carrying the TetR/Pip OFF repressible system (WT-pFRA42B), using an adaptation of the method developed by Boldrin *et al.* [24] (figure 1*a*). The correct recombination event leading to the replacement of P*fas* by the regulated promoter P*ptr* was confirmed by genomic PCR (figure 1*b*). In the resulting conditional mutant strain, named *fas* cKD, expression of *fas* is downregulated by the addition of anhydrotetracycline (ATc) to the culture media, which leads to a partial depletion of the multi-domain FAS I. When this strain was plated on 7H10 solid medium containing 200 ng ml^−1^ ATc, no growth was detected, suggesting that the repression of *fas-acpS* expression from P*ptr* was stringent enough to prevent FAS levels compatible with cell viability (electronic supplementary material, figure S2). Growth of the parental strain WT-pFRA42B was unaffected by the presence of ATc at this concentration (electronic supplementary material, figure S2).

**Figure 2.**
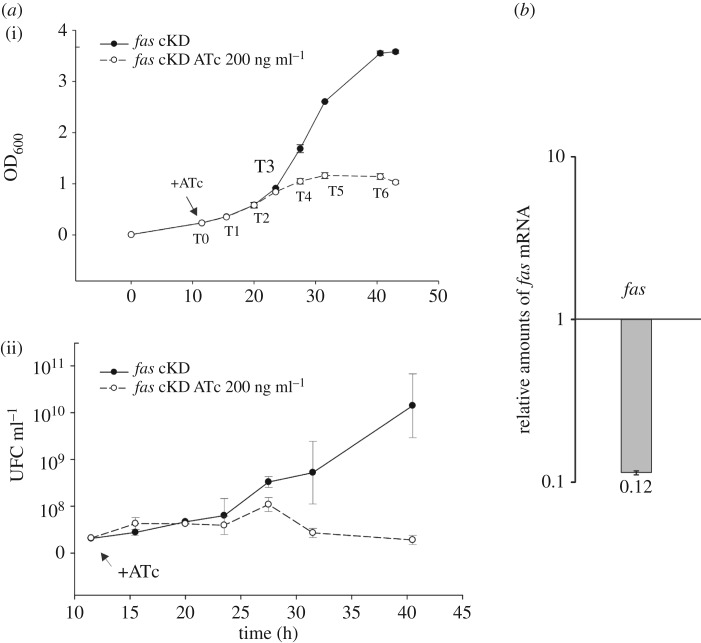
Growth curve and cell viability of the *M. smegmatis* conditional mutant strain *fas* cKD. (*a*) After 12 h of growth in 7H9 medium, the culture was divided into two equal fractions and one of them was supplemented with ATc 200 ng ml^−1^ (indicated with an arrow). Growth was followed by measuring OD_600_ (i). Cell viability was assayed at different time points (T0 to T6) by plating dilutions of the culture on 7H10 medium and then counting the number of colony forming units (CFU ml^−1^) (ii). (*b*) Changes in the relative amounts of *fas* mRNA measured by quantitative RT-PCR. Values represent the mean difference between *fas* cKD strain grown with and without ATc 200 ng ml^−1^, and are normalized using *sigA* as an invariant transcript. Samples for RNA extraction were collected 12 h after addition of ATc (T3).

To study the effect of FAS depletion at different growth stages, the conditional mutant strain *fas* cKD was grown in 7H9 medium in the absence and in the presence of ATc 100 or 200 ng ml^−1^. As depicted in electronic supplementary material, figure S2*c*, growth of the *fas* cKD strain was inhibited when ATc was added at the same time of the inoculum. Therefore, in order to find a condition where we could explore the impact of *fas* depletion in lipid metabolism, ATc was added at different time points after inoculation of the culture. Electronic supplementary material, figure S2, shows that in the absence of ATc, the mutant strain exhibited a typical exponential growth curve, comparable with the isogenic strain WT-pFRA42B; however, when ATc was added at the beginning of the exponential phase, at OD_600_ ≤ 0.2 (T0), cells started to clamp and grow at a lower rate to finally stop dividing at OD_600_ of approximately 0.8–1 (figure 2*a*). Exogenous FA supplementation (palmitic acid, oleic acid or a mix of them) was not able to restore growth of the *fas* cKD mutant after the addition of ATc (data not shown). When cell viability, determined as the number of colony forming units (CFU), was analysed before and after addition of ATc 200 ng ml^−1^, the *fas* cKD strain showed a reduction of 31% in CFU numbers 12 h after the addition of ATc (figure 2*a*), thus confirming the essentiality of *fas* for the survival of *M. smegmatis* in axenic cultures.

In order to correlate cell growth arrest to the expression level of *fas*, the relative amount of *fas* mRNA was measured by quantitative RT-PCR after 12 h of exposure to ATc, before cells stopped growing and viable counts decreased (T3). Relative amounts of *fas* mRNA in *fas* cKD were compared in cell cultures grown in the presence or absence of ATc, representing the physiological level of *fas* mRNA (figure 2*b*). In the presence of ATc, the levels of *fas* mRNA in the *fas* cKD strain decreased dramatically (approx. 90%) compared with the levels found in the absence of ATc. In addition, it is worth noting that the level of *fas* mRNA in the parental strain WT-pFRA42B was not affected by the presence of ATc in the medium (data not shown).

### 3.2. Knock down of *fas* expression results in a severe impact on FAS I but not on FAS II activity

**Figure 3.**
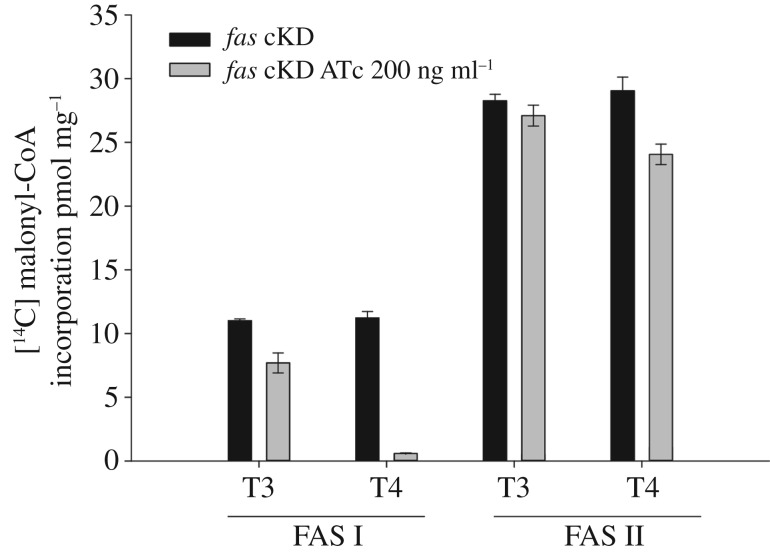
Determination of *in vitro* FAS I and FAS II activities. Cell-free extracts were prepared from *fas* cKD cultures grown in the absence or presence of ATc 200 ng ml^−1^ at T3 and T4. FAS I and FAS II activities were measured in a reaction containing 1 mg of cell-free protein extracts in the presence of [2-^14^C] malonyl-CoA as extender unit and acetyl-CoA or palmitoyl-CoA as FAS I or FAS II starter units, respectively. After incubation and extraction of saponifiable lipids, the incorporated radioactivity was measured. The results are the mean values and standard deviation of three independent biological replicates. All results were normalized by OD_600_ and total protein concentration.

To determine the physiological consequences associated with decreasing *fas* expression, samples from cultures of the *fas* cKD strain grown with and without ATc 200 ng ml^−1^ were collected at different time points (T3 and T4), and cell-free extracts were prepared for the determination of FAS I and FAS II activity. As observed in figure 3, in the presence of ATc the activity of the FAS I system was 25% lower at T3 and barely detected after 24 h of incubation (T4) when compared with the same enzymatic activity determined in the protein extracts the *fas* cKD cultures grown without ATc. Surprisingly, when the same cell-free extracts were used to analyse FAS II activity, we found that this enzyme activity was unaffected at T3 in the cultures grown with ATc and showed only a slight reduction (17%) at T4, probably due to a pleiotropic effect on cell metabolism triggered by the strong depletion of FA biosynthesis (electronic supplementary material, figure S3).

In order to determine the levels of metabolic activity in the *fas* cKD strain, we performed [^3^H] leucine labelling experiments at different time points after the addition of ATc 200 ng ml^−1^ and compared with the culture grown without ATc. As shown in electronic supplementary material, figure S3, the metabolic activity of the mutant cells was comparable with that of the control strain for about 12 h after the addition of ATc. However, at the time the cells stop dividing (T4), we observed a 35% reduction of [^3^H] leucine incorporation, suggesting a decrease in cell viability.

**Figure 4.**
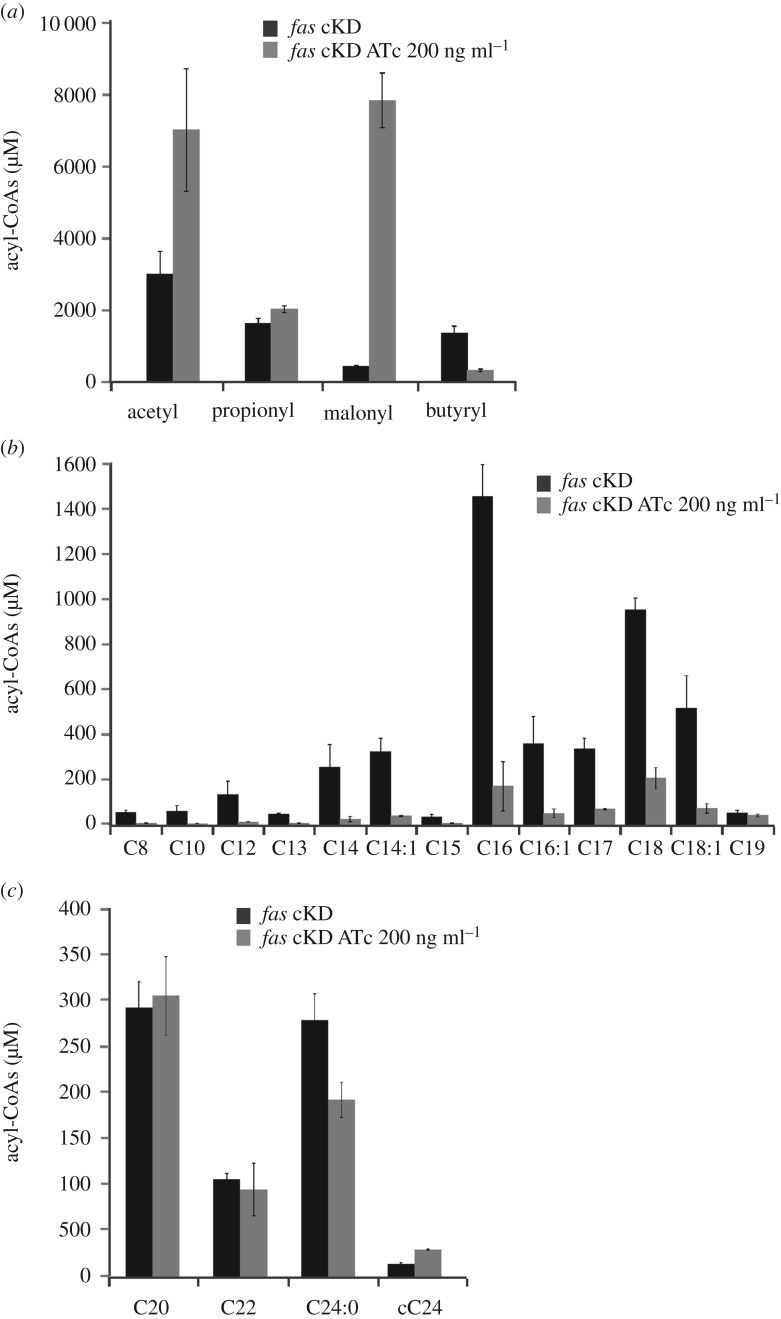
Acyl-CoAs pool analysis in *fas* cKD. Acyl-CoAs were extracted from cultures incubated in the absence or presence of ATc 200 ng ml^−1^ at T3. Samples corresponding to the same number of cells were analysed by LC-MS. (*a*) Short-chain acyl-CoAs. (*b*) Medium-chain acyl-CoAs. (*c*) Long-chain acyl-CoAs. Results are the means of three independent experiments ± standard deviations (*n* = 3). cC_24_: carboxy-C_24_-CoA.

To further analyse the consequences of a reduced FAS I activity in lipid metabolism, we determined the acyl-CoA composition in the *fas* cKD mutant strain in the presence or absence of ATc. Acyl-CoAs extracted from a fixed number of cells were analysed by LC-MS as previously described [31]. As shown in figure 4*a*, an increase in the content of acetyl-CoA and malonyl-CoA was observed in the cultures grown in the presence of ATc. This result is consistent with an accumulation of these metabolites as a consequence of a reduced FAS I activity under these growth conditions. Furthermore, the lower levels of FAS I activity strongly impacted on the synthesis of the complete repertoire of medium-chain-length acyl-CoAs (figure 4*b*). Surprisingly, the levels of the long-chain acyl-CoAs synthesized by the mutant showed no differences for C_20_-CoA and C_22_-CoA, and a relatively small difference (25%) for C_24_-CoA (figure 4*c*). Moreover, the levels of α-carboxy-C_24_-CoA, a key intermediate for MA biosynthesis, showed increased levels in the ATc treated cultures (figure 4*c*), confirming that the levels of C_24_-CoA were less affected than those corresponding to the medium-chain acyl-CoAs.

**Figure 5.**
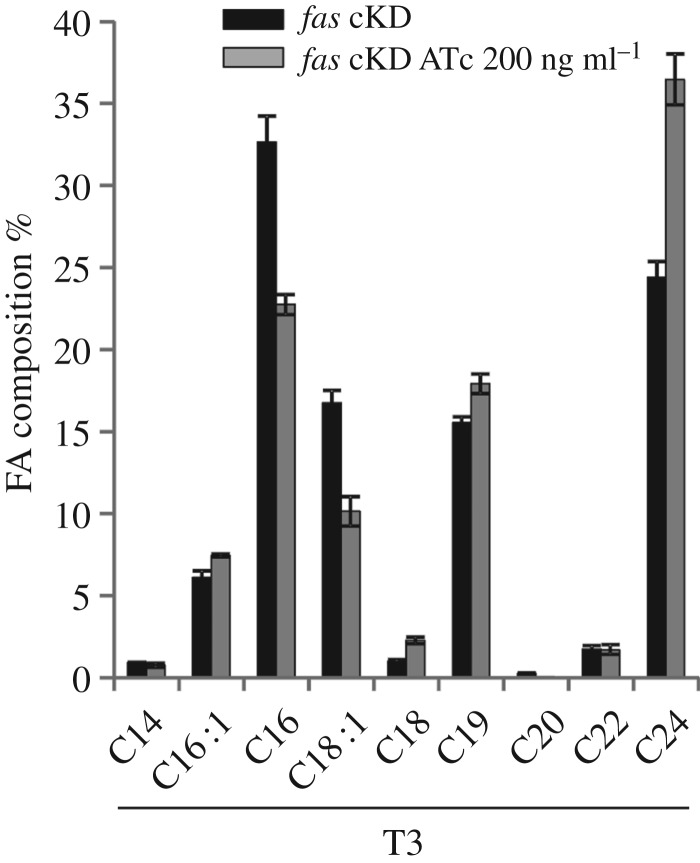
Relative fatty acid composition of *fas* cKD. The mutant strain was grown in 7H9 in the absence or presence of ATc 200 ng ml^−1^. At T3 fatty acids hydrolysed from total lipids were extracted from the same number of cells and analysed by GC-MS. Results are the means of three independent experiments ± standard deviations (*n* = 3).

The FA composition of the *fas* cKD mutant strain grown in the presence or absence of ATc was analysed by GC-MS. As shown in figure 5 and electronic supplementary material, figure S4, the relative composition of FA changed upon the addition of ATc: the levels of medium-chain-length FA were reduced approximately 10% with the concomitant increase (approx. 12%) of the long-chain C_24_. These results are in agreement with the differences observed in the acyl-CoA content of the mutant strain.

### 3.3. Loss of FAS I activity differentially affected the de novo FA and MA biosynthesis

**Figure 6.**
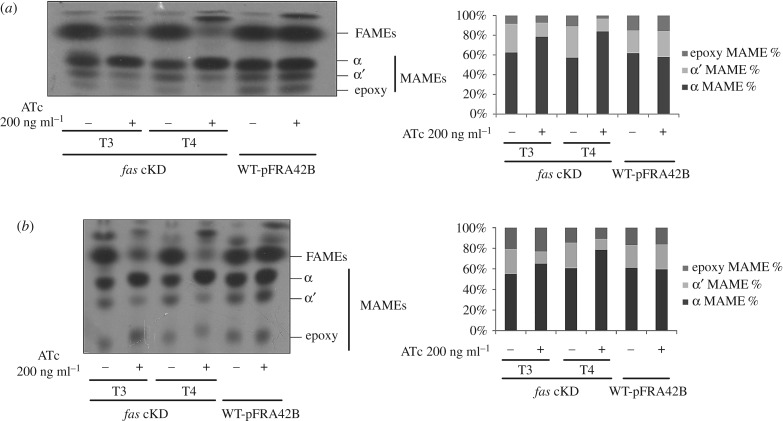
De novo fatty acid and mycolic acid biosynthesis. Cells from *fas* cKD and WT-pFRA42B cultures, grown in the presence or absence of ATc 200 ng ml^−1^, were labelled with [1-^14^C] acetate at T3 and T4 for 1 h at 42°C. After organic extraction, ^14^C-labelled methyl esters of mycolic acids (MAMEs) and fatty acids (FAMEs) were separated by thin layer chromatography. Optical density standardization was performed during the methyl esters extraction step. (*a*) Solvent system: hexane: ethyl acetate (9 : 1 v/v). (*b*) Solvent system: dichloromethane. Quantification of the radiolabelling intensity is shown for both TLC.

To study the impact of FAS I depletion in the de novo synthesis of FA and MA, [^14^C] acetate labelling experiments were carried out with the *fas* cKD mutant strain. Cultures were grown with and without ATc 200 ng ml^−1^ and their lipid content analysed by radio-TLC. As shown in figure 6, de novo synthesis of FA was progressively and significantly reduced after 12 and 16 h exposure to ATc (T3 and T4, respectively). Remarkably, MA content did not decrease at these time points, although there were differences between the *fas* cKD mutant strain grown with and without ATc with regard to the relative abundance of the three classes of MAMEs (figure 6*a*). The relative percentages for α-, α′- and epoxy-mycolates from the *fas* cKD strain grown without ATc were 60, 30 and 10%, respectively (figure 6*a*). Similar profiles were obtained for the WT-pFRA42B isogenic strain grown with and without ATc. The percentages for the mutant strain *fas* cKD grown with ATc 200 ng ml^−1^ were 80, 14, 7% and 85, 12, 3% in T3 and T4, respectively, showing a significant increase in the abundance of α-mycolates (25% in T4) with a compensatory reduction of α′-mycolates and epoxy-mycolates (figure 6). In addition to this alteration, in the presence of ATc the TLC mobility of each of the MA species showed a slight but reproducible increment, compared with the mutant strain grown without ATc, suggesting the prevalence of MA of longer carbon chain lengths. When the same samples were run under different solvent conditions, in order to improve the separation of the different species of mycolates, the *fas* cKD strain grown with ATc clearly showed a different distribution of MA species and all of them had a bigger Rf compared with the ones extracted from the cultures grown without ATc or from the isogenic strain WT-pFRA42B (figure 6*b*).

### 3.4. Lipidomics of the *fas* cKD mutant

**Figure 7.**
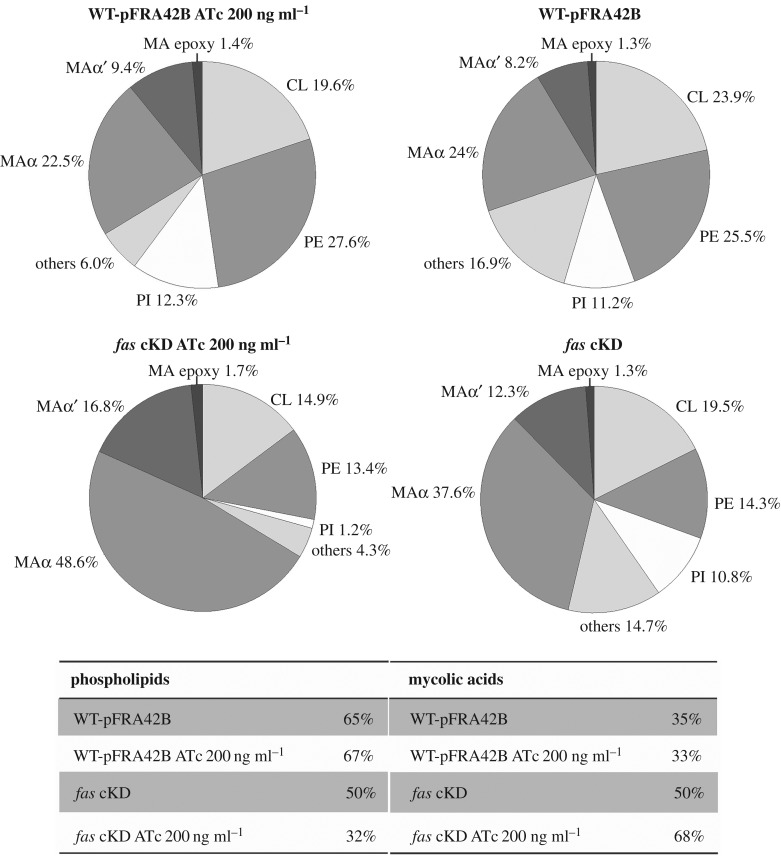
LC-MS analyses of the phospholipids and mycolic acids present in the *fas* cKD mutant and in the isogenic strain WT-pFRA42B. The values indicated represent the relative abundance of the MS signals corresponding to the different lipid classes found in the samples. Results are the means of three independent experiments. Others: PG, PA, lysoPE, lysoPI, lysoPG, lysoPA and mycolilphospholipids.

Based on the previous results showing important qualitative and quantitative modifications on the acyl-CoA pools and on the FA and MA contents of the *fas* cKD mutant strain, compared with the isogenic WT-pFRA42B strain, we decided to study in more detail the impact of low FAS I activity levels on the global lipid profile. For this, we performed comparative lipidomic analyses between the conditional *fas* mutant and its isogenic strain. Briefly, we extracted cell-associated lipids from the *fas* cKD mutant strain grown in the absence and in the presence of ATc (T3) and analysed the total lipid content by high-performance liquid chromatography–mass spectrometry (HPLC-MS). As a control, we extracted and analysed lipids from the isogenic strain WT-pFRA42B grown in the same conditions. Figure 7 shows the results obtained for the lipids analyses carried out in and negative ion mode. The most significant differences in the content of MA between the wild-type and mutant strains grown in the presence or absence of ATc were those found for the relative composition of α, α′ and epoxy mycolates, as had been previously observed in the radio-TLC, and in the size of the molecules, on average 1–2 carbons longer in the mutant with reduced levels of FAS I activity (figure 7; electronic supplementary material, figure S5*a*,*b*). Furthermore, tandem mass spectrometry analysis (MS-MS) of the individual types of mycolates showed that α-branches of the mycolates from both control WT-pFRA42B and *fas* cKD mutant grown in the absence and in the presence of ATc contained mainly 24 carbons, indicating that the additional one to two carbon units present in the MA of the *fas* mutant are in the merochains (electronic supplementary material, figure S5*c*). In addition, lipids bearing MA moieties, such as glycerol monomycolates (GroMM) or trehalose monomycolates (TMM), detected in the positive mode, also showed increased acyl chain length (electronic supplementary material, figure S6). On the other hand, the relative amount and composition of PL was also severely modified. For instance, the WT-pFRA42B isogenic strain has a relative percentage of PL and MA of approximately 65 and 35%, respectively. This relationship is completely the opposite in the mutant strain grown with ATc (figure 7), reflecting the strong impact in FA and therefore in PL biosynthesis when *fas* expression is reduced. The relative composition of PL showed a more biased effect towards phosphatidylinositol (PI), which was significantly reduced in the mutant strain under low levels of FAS I (figure 7). The acyl-chain substitutions of cardiolipins (CL) and phosphatidylethanolamine (PE) also proved to be longer in the mutant strain grown in the presence of ATc (electronic supplementary material, figure S7*a*,*b*). Furthermore, MS-MS of unusually long PE species, which were only found in the mutant strain grown in the presence of ATc, showed the presence of acyl chains substitutions of 24 carbon atoms in their structure (electronic supplementary material, figure S7*c*). Another relevant change observed in the qualitative composition of the PL was the significant increase in the double bonds of the acyl residues, most significant being those of the CL, phosphatidylglycerol (PG) and PI (electronic supplementary material, figure S8). Analysis carried out in the positive mode was more difficult in terms of the relative content of each of the molecular species identified, such as diacylglycerol (DAG), monomeromycolyl DAG (MMDAG), GroMM, TMM, phosphatidylinositol mannoside (PIM) and TAG, due to the high content of the latter compound in the samples.

### 3.5. Central role of triacylglycerides in the sustained synthesis of mycolic acids in the *fas* cKD mutant

**Figure 8.**
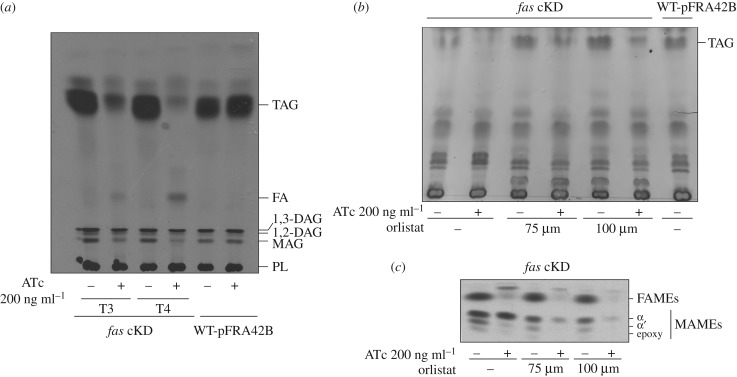
Inhibition of TAG hydrolysis limits de novo MA biosynthesis in the FAS I knock down mutant. (*a*) De novo biosynthesis of TAG is severely reduced once *fas* transcription is shut down. Aliquots containing the same number of cells from *fas* cKD cultures grown with and without ATc 200 ng ml^−1^ were labelled with [1-^14^C]-acetate at T3 and T4 for 1 h at 42°C. Total lipids were extracted and then analysed on silica gel TLC plates developed in hexane/diethylether/acetic acid (75 : 25 : 1, v/v/v). As control, the same number of cells of a WT-pFRA42B culture were labelled and lipids were extracted and developed in the same TLC. (*b*) Treatment with tetrahydrolipstatin (Orlistat) reduced TAG hydrolysis. *fas* cKD cultures were grown with or without ATc 200 ng ml^−1^, and in the presence or absence of Orlistat 75 and 100 µM. Culture aliquots containing the same number of cells were centrifuged at T3 for total lipid extraction followed by analysis on silica gel TLC plates developed in hexane/diethylether/acetic acid (75 : 25 : 1, v/v/v). Total lipids extracted from WT-pFRA42B were loaded as control. Chemical staining with Cu-phosphoric stain was used for detection. (*c*) Reduced TAG hydrolysis upon treatment with tetrahydrolipstatin (Orlistat) limits the de novo mycolic acid biosynthesis. Aliquots from *fas* cKD cultures grown with or without ATc 200 ng ml^−1^, and in the presence or absence of Orlistat 75 and 100 µM, were labelled with [1-^14^C]-acetate at T3 for 1 h at 42°C. The ^14^C-labelled methyl esters of mycolic acids (MAMEs) and fatty acids (FAMEs) were extracted and analysed on silica gel TLC plates developed in hexane/ethyl acetate (9 : 1 v/v). Aliquots extracted from the same number of cells for each sample were loaded.

One of the most intriguing results of the studies carried out with the *fas* cKD mutant strain was the continuous synthesis of MA at T3 and T4 in the presence of ATc, when the cells are deprived of most of the FAS I enzyme and when the de novo FA biosynthesis appears strongly compromised (figure 6). Considering that FAS I provides the acyl-CoAs that are further elongated by FAS II with malonyl-ACP to synthesize the meromycolic acids, it was expected that MA biosynthesis would follow the same detrimental impact as the de novo FA biosynthesis in the ATc-treated cultures. Therefore, in order to find out the source of acyl-CoAs under these conditions we analysed the impact of the reduction of FAS I activity in the de novo TAG biosynthesis. For this, cultures of the *fas* cKD mutant strain were grown with and without ATc 200 ng ml^−1^, labelled with [^14^C] acetate and their TAG content analysed by TLC. As shown in figure 8*a*, de novo synthesis of TAG was progressively reduced after 12 and 16 h exposure to ATc (T3 and T4, respectively). The TLC also showed an accumulation of free FA that increased throughout time. This result could suggest that besides a reduced TAG biosynthesis, the cells started to actively degrade the accumulated ones in order to compensate the lack of FAS I activity and provide FA to be used in other metabolic pathways (e.g. in MA biosynthesis). This hypothesis was tested treating the cultures with tetrahydrolipstatin, an inhibitor of lipases marketed as Orlistat, which was previously shown to inhibit TAG hydrolysis in mycobacteria [32]. As shown in figure 8*b*, total TAG content in the *fas* cKD strain grown in the presence of ATc and Orlistat is higher than in the strain grown in the presence of ATc but in the absence of Orlistat, and this difference is bigger when higher concentrations of Orlistat are used, suggesting that the inhibitor is clearly having an inhibitory effect in the lipase/s involved in TAG hydrolysis. Moreover, [^14^C] acetate labelling experiments with the *fas* cKD mutant strain treated with Orlistat revealed a clear reduction in MA biosynthesis when the cultures were grown with ATc (figure 8*c*), suggesting that MA biosynthesis relies in TAG hydrolysis when the acyl-CoAs produced by the FAS I system become limited.

### 3.6. Cross-talk between the FAS I and FAS II systems

**Figure 9.**
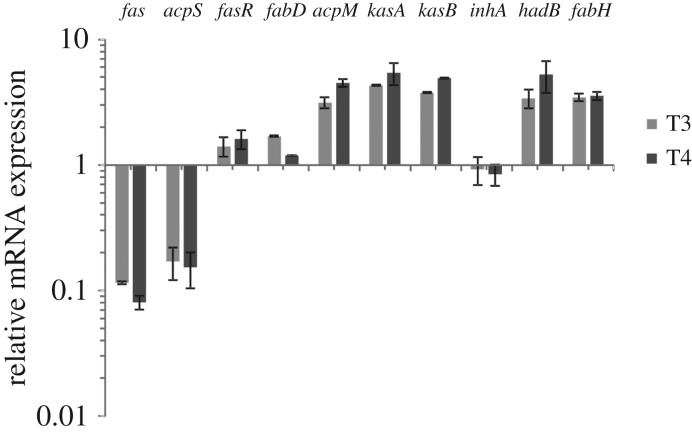
The reduced expression of *fas* in the *fas* cKD mutant strain leads to a global upregulation of genes required for mycolic acid biosynthesis. The relative expression profile of *fas, acpS, fasR, fabD, acpM, kasA, kasB, inhA, hadB* and *fabH* was determined by qRT-PCR at T3 and T4. The cycle threshold (CT) value of each gene of interest was normalized by using the housekeeping gene *sigA*. Values represent the mean difference between the *fas* cKD strain grown with and without ATc 200 ng ml^−1^. The quantification was performed in triplicate ± standard deviation.

*Mycobacterium* has an extremely complex lipid metabolism. In order to preserve lipid homeostasis it is essential for these bacteria to maintain a tight regulation between the FAS I and FAS II components. Therefore, to unveil the cross-talk between these two systems and to understand the response of the FAS II genes to the knock down of *fas* expression, we performed a quantitative RT-PCR experiment with RNAs extracted from the *fas* cKD strain grown in the presence or absence of ATc. As shown in figure 9, cultures grown in the presence of ATc showed a markedly reduced expression of the *fas-acpS* operon, 90% lower expression for the *fas* gene and 85% for *acpS* than that found in the absence of ATc. The higher level of expression observed for the transcriptional activator of the *fas-acpS* operon, *fasR*, is probably a response to try to keep *fas* and *acpS* expression at their normal levels. The fact that MA biosynthesis remained active after the treatment of the mutant with ATc suggested that the MA biosynthesis genes should be actively transcribed in this conditions. Indeed, as shown in figure 9, the complete *fasII* operon genes (*fabD-acpM-kasA-kasB*) showed an increased level of transcription in the mutant strain treated with ATc. Moreover, the expression level of *fabH*, a key component that links FAS I and FAS II activities, was also higher upon treatment of the *fas* cKD strain with ATc (figure 9).

## 4. Discussion and conclusion

Mycobacterial survival depends on membrane lipid homeostasis and on the ability to adjust lipid composition to adapt the bacterial cell to different environments. This is especially relevant for *M. tuberculosis*, a pathogen with a very complex lifestyle capable of modulating its metabolism in response to the environmental changes within the host, where several components of the unusual lipid-rich cell wall of this organism play a key role in the outcome of the infection [33]. Therefore, understanding the interaction between FAS I and the metabolic pathways that rely on FAS I products to synthesize the complex cell wall lipids, as well as the membrane PL and the storage compounds TAG, is a key step to better understand how lipid homeostasis is regulated in mycobacteria.

In order to gain information regarding this critical regulatory process of lipid metabolism in mycobacteria, we initiated this study by constructing a conditional mutant in the *fas-acpS* operon of the model organism *M. smegmatis*. The genetic and physiological characterization of this mutant showed that FAS I is a housekeeping component of the mycobacteria lipid metabolism and that the absence of this function cannot be compensated *in vitro* by the addition of FA to the culture medium. This result is consistent with those obtained for Gram-negative and most Gram-positive bacteria, where the inhibition of de novo FA biosynthesis by specific FAS II inhibitors cannot be overcome by supplementing FA to the media or by the host lipids in infection models [34,35].

The conditional *fas* cKD mutant showed a neat growth response to the addition of ATc. The mutant strain significantly slowed down its growth rate 12 h after the addition of ATc and stopped dividing soon after. This growth defect is well correlated with the lower expression of *fas* and the concomitant and specific reduction of FAS I activity in the first hours after ATc treatment. As could be expected, the lower levels of FAS I provoked an accumulation of its substrates, acetyl-CoA and malonyl-CoA, and a strong reduction on the absolute levels of the C_12_-C_18_ acyl-CoAs. Interestingly, the impact of the reduced FAS I activity on the levels of the long-chain acyl-CoAs (C_19_ to C_24_) was rather weak. It is well established that FAS I exhibits a bimodal product behaviour and that the two dominant products are C_16-18_-CoA and C_24_-CoA in *M. smegmatis*. However, it has also been demonstrated that the overall rate of FA biosynthesis as well as the product distribution of this enzyme becomes affected by endogenous polysaccharides or by interactions of the mycobacterial FAS I with other FA processing enzymes like the FAS II system [25,36–38]. Thus, the unexpected accumulation of long-chain acyl-CoAs, when FAS I was downregulated, could be the result of a biochemical change in the bimodal behaviour of the FAS I system when the cells become stressed by the deficiency of *de novo* FA biosynthesis. Alternatively, the accumulation of long-chain acyl-CoAs could also represent a differential degradation of the different chain-length acyl-CoAs through the β-oxidation cycle. The relative composition of total FA in the *fas* cKD mutant also shows a bias towards the long-chain FA, being the most abundant C_24_ FA under conditions of *fas* downregulation, suggesting that TAG and PL may have been enriched in long acyl chains.

A very intriguing result was the continuous synthesis of MA at T3 and T4 of the growth curve, where de novo FA biosynthesis appears severely compromised. In fact one would expect that the inhibition of de novo FA biosynthesis would also result in the inhibition of MA synthesis, because the FAS II system and the long-chain acyl-CoA carboxylase 4 utilize the products of FAS I as substrates of their corresponding reactions to generate the meromycolic acid and the carboxy-C_24_-CoA, respectively, before the final step of condensation mediated by Pks 13 [39]. Therefore, to solve this puzzle and to seek for the possible source(s) of these precursors we inhibited the degradation of TAG with Orlistat, a strong lipase inhibitor. The results showed that the inhibition of TAG degradation impaired, at least partially, de novo synthesis of MA under conditions of FAS I reduction, suggesting that FA released from TAG can compensate, to some extent, for the absence (or reduced levels) of de novo FA synthesized by FAS I. It has long been known that under starvation conditions mycobacteria depend on TAG mobilization for energy production [40]. However, to our knowledge, this is the first time that TAG are experimentally demonstrated to be a reservoir of FA as precursors for the biosynthesis of more complex molecules in bacteria.

A detailed analysis by LC-MS of the MA species synthesized at late time points under conditions of low levels of FAS I revealed that α MA not only became more abundant with respect to the other species, α′ and epoxy, but also longer on average. This result suggests that FAS II probably loses its natural regulation either because of the absence of substrate competition by FAS I or due to other more complex regulatory processes. In fact, it was noticeable that almost all the FAS II encoding genes, with the exception of *inhA*, were upregulated as a response to *fas* repression. This condition is similar to that where KasA is overexpressed and where the outcome was the synthesis of longer molecules of meromycolic acids [41,42]. The low levels of FAS I activity provoked a clear unbalance on the relative composition of the acyl-CoAs pool, which not only are the precursors for the biosynthesis of other complex lipids but also the signal molecules sensed by transcriptional regulators of lipid metabolisms in several species [43–46], including FasR of *M. smegmatis*, the positive regulator of the *fas-acpS* operon [16]. Moreover, we recently found that these effector molecules are also sensed by MabR, the transcriptional regulator of the *fasII* operon of mycobacteria (to be published elsewhere). Therefore, by manipulating the expression of *fas* to induce a conditional depletion of FAS I we showed that not only the de novo synthesis of FA is affected, but that this metabolic stress produces a strong and pleiotropic effect on the regulation of other lipid related genes (lipid homeostasis) that inevitably should lead to cell death. In previous work, we also showed that a modification of the expression levels of the *fas-acpS* or the *fasII* transcriptional regulators, *fasR* and *mabR*, respectively, also provoked a loss of the natural cross-regulation between the FAS I and FAS II systems [16,17].

A detailed lipidomic analysis of the *fas* cKD mutant strain showed some remarkable features besides the ones related to the MA that were discussed above. LC-MS analysis of the PL synthesized under conditions of *fas* repression revealed a strong reduction on the relative composition of PI, which dropped almost four-fold compared with the *fas* cKD strain growing without ATc or the isogenic strain. PI and its metabolically derived products such as PIM and linear and mature branched lipomannan are prominent PL/lipoglycans of *Mycobacterium* sp. and have been shown to be essential for cell viability [47]. The preferential impact in the biosynthesis of this PL is intriguing and might be related to the regulation of the PI synthase. When analysing the length of the acyl moieties esterified to the different PL of the mutant, it was noticeable that only PE and CL contained C_24_ FA. These FA were not present in the PL of either the mutant strain grown in the absence of ATc or the isogenic strain WT -pFRA42B. Another interesting feature that came out of this analysis was that the FA present in the PL of the ATc treated mutant contained higher levels of unsaturation. This phenomenon might occur to mitigate the rigidity of the plasma membrane imparted by the presence of long-chain FA in the PL [48].

A number of studies indicate that *M. tuberculosis* utilizes a restricted set of host-derived nutrients to persist within its host cell. Most notably, FA and cholesterol are important nutrients during infection *in vivo* or in infected macrophages in cultures [49]. Developing a conditional *fas* mutant in *M. tuberculosis* will help to define the essentiality of de novo FA biosynthesis in the survival of this pathogen during infection, and in that way it should help to unambiguously establish if FAS I is a good target for drug development. Furthermore, a conditional mutant of FAS I in *M. tuberculosis* will also help us to study how the complex regulatory network that links the different lipid-related pathways in this deadly pathogen responds to or tries to compensate for a deficiency of de novo synthesized FA.

## Supplementary Material

Supporting Information

## Supplementary Material

Tables S1-S2-S3

## Supplementary Material

Figuras S1-S8
