# Supplementary material for: A conditional mutant of the fatty acid synthase unveils unexpected cross talks in mycobacterial lipid metabolism
Source: Open Biol. 2017 Feb 22;7(2):160277. doi: 10.1098/rsob.160277 (PMC5356441; doi:10.1098/rsob.160277)
Supplement: Supporting Information [file rsob160277supp1.docx]

**SUPPORTING INFORMATION**

**S1 Table. Plasmids used in this work.**

**S2 Table. Strains used in this work.**

**S3 Table. RT primers used in this study.**

**FIGURE LEGENDS TO SUPPLEMENTARY FIGURES**

**Fig S1. Schematic representation of lipid biosynthesis in mycobacteria.**

**Fig S2. Construction and analysis of the *fas* cKD mutant.** (A) The wild type strain *M. smegmatis* mc^2^ 155 (WT), the *fas* cKD mutant and the isogenic strain WT-pFRA42B were plated on Middlebrook 7H10 medium with or without 200 ng/ml ATc. (B) Growth curve of the isogenic strain WT-pFRA42B in the absence (filled circles) and in the presence of ATc 200 ng/ml added at T1 (empty circles). (C) Growth curve of *fas* cKD in the presence of different concentrations of ATc. *fas* cKD was grown at 42 °C in Middlebrook 7H9 in the absence of ATc (filled circles), in the presence of 100 ng/ml ATc added at T0 (empty circles) or T1 (empty triangles) and in the presence of 200 ng/ml ATc added at T0 (filled triangles) or T1 (filled squares). Growth was followed by measuring optical density at 600 nm (OD_600_).

**Fig S3.** **Determination of metabolic activity by incorporation of [^3^H] leucine in axenic cultures**. Cultures of *fas* cKD grown in the absence and presence of ATc 200 ng/ml were labeled for 1h with 0.5 µCi/ml of L-[4,5 ^3^H (N)] leucine at 42°C at different time points as indicated in the growth curve (T1 to T6). Cells were centrifuged, washed three times with 10 mM Tris-HCl, pH 8 and resuspended in 1 ml scintillation liquid. The radioactivity incorporated into the cells was measured in a Beckman scintillation liquid counter. The results are the mean of three independent experiments ± standard deviations (n = 3). All results were normalized by OD_600_. * P < 0.001

**Fig S4. Relative fatty acid composition of *fas* cKD.** The *fas* cKD strain was grown in the absence and presence of ATc 200 ng/ml. Fatty acids hydrolyzed from total lipids were extracted from the same number of cells at T3 and T4 and analyzed by GC-MS. WT-pFRA42B was used as control.

**Fig S5.** **Detailed analysis of mycolic acid molecular species.** (A) The table shows the weighted average of total number of carbon atoms per molecule within the population of free α MA of the different samples. The results are the mean of three independent experiments ± standard deviations (n = 3). The *P* value was calculated as unpaired *t* test with Welch's correction between *fas* cKD and *fas* cKD ATc 200 ng/ml. (B) The histogram shows the distribution of the number of carbon of free MA α in *fas* cKD grown with and without ATc 200 ng/ml. (C) Tandem mass spectra of the α MA with 81 (1178.21 *m*/z) carbon atoms in three different samples showing the C24 α branch (367.35 *m*/z) signal. The inset shows where the fragmentation is produced in the MA structure.

**Fig S6.** **Detailed analysis of mycolic acid molecular species.** A) The table shows the weighted average of total number of carbons atoms within the entire population of alpha TMM and alpha GroMM of the different samples. The results are the mean of three independent experiments ± standard deviations (n = 3). The *P* value was calculated as unpaired *t* test with Welch's correction between *fas* cKD and *fas* cKD ATc 200 ng/ml. B) The histogram shows the distribution of the number of carbon of alpha TMMs main species in *fas* cKD grown with and without ATc 200 ng/ml. C) The histogram shows the distribution of the number of carbon of alpha GroMMs main species in *fas* cKD grown with and without ATc 200 ng/ml.

**Fig S7.** **Detailed analysis of phospholipid molecular species.** (A) The table shows the weighted average of total number of carbon atoms per molecule within the population of phospholipids of the different samples. The results are the mean of three independent experiments ± standard deviations (n = 3). The *P* value was calculated as unpaired *t* test with Welch's correction between *fas* cKD and *fas* cKD ATc 200 ng/ml. (B) The histogram shows the distribution of the number of carbon atoms of phosphatidylethanolamine (PE) in *fas* cKD grown with and without ATc 200 ng/ml. (C) Tandem mass spectra of the unsaturated PE with 42 carbon atoms (828.64 *m*/z) in *fas* cKD grown with ATc 200 ng/ml showing the presence of the signal corresponding to the C_24_ fatty acid (367.35 *m*/z). The inset shows where the fragmentation is produced in the PE structure.

**Fig S8.** **Saturation level of the fatty acid substituents of the major phospholipids present in the *fas* cKD mutant.** Histograms represent the levels of unsaturation of the fatty acids constituents of cardiolipin, phosphatidylinositol, phosphatidylglycerol and phosphatidylethanolamine. Results are the means of three independent experiments. Asterisks correspond to a *P* value ˂ 0.05 between *fas* cKD grown in the presence of ATc 200 ng/ml and the other samples analyzed (*fas* cKD grown in the absence of ATc 200 ng/ml and WT-pFRA42B grown in the presence or absence of ATc 200 ng/ml). The *P* value was calculated as ANOVA with Tukey's multiple comparisons test.
