# Supplementary material for: A conditional mutant of the fatty acid synthase unveils unexpected cross talks in mycobacterial lipid metabolism
Source: Open Biol. 2017 Feb 22;7(2):160277. doi: 10.1098/rsob.160277 (PMC5356441; doi:10.1098/rsob.160277)
Supplement: Tables S1-S2-S3 [file rsob160277supp2.docx]

| **Plasmids** | **Description** | **Ref.** |
| --- | --- | --- |
| **pFR42** | pMP349 derivative carrying the 5’ region of the *M. smegmatis* *fas* gene (Rv2524c) under the control of P*_ptr_* (P*_ptr_* -5´*fas* MS), Apra^r^ | This study |
| **pFR18** | pPR27 derivative carrying P*_ptr_* -5´*fas* MS, Gm^r^ Apra^r^ | This study |
| **pFRA42B** | pFRA40 derivative; P*smyc*-*tetR*(→); P*furA102 tetOpip*(→)*;*P*ptr-lacZ*; *int*; Str^r^ | [1] |
| **pFRA50** | pSM240 derivative carrying P*_ptr_*, Hyg^r^ | [1] |
| **pMP349** | *E. coli*-*Mycobacterium* shuttle vector, Apra^r^ | [2] |
| **pPR27** | *E. coli-Mycobacterium* shuttle vector, *oriM* temps, *sacB, xylE,* Gm^r^ | [3] |

**Table S1. Plasmids used in this work.**

Gm^r^*,* gentamicin resistance*;* Km^r^, kanamycin resistance; Apra^r^, apramycin resistance; Str^r^, streptomycin/ spectinomycin resistance; Hyg^r^, hygromycin resistance.

**Table S2. Strains used in this work.**

| **Strain** | **Comments** | **Ref.** |
| --- | --- | --- |
| *Escherichia coli* DH5α | *E. coli K12 F- ΔlacU169 (φ80lacZΔM15) endA1 recA1hsdR17 deoR supE44 thi-1λ- gyrA96 relA1* | [4] |
| *Mycobacterium smegmatis* mc^2^155 | Electroporation-proficient *ept* mutant of *M. smegmatis* strain mc26 | [5] |
| WT-pFRA42B | *M. smegmatis* mc^2^*155* harboring pFRA42B, Sm^r^ | This study |
| MSpFRA42B pFR18 | *M. smegmatis* mc^2^*155* harboring pFRA42B and pFR18, Am^r^, Sm^r^ | This study |
| *fas* cKD | *M. smegmatis fas* mutant strain harboring pFR18 integrated into *fas* locus and pFRA42B, Am^r^ , Sm^r^ | This study |

Am^r^, apramycin resistance; Sm^r^, streptomycin/spectinomycin resistance.

**REFERENCES**

1. Boldrin, F., Casonato, S., Dainese, E., Sala, C., Dhar, N., Palù, G., Riccardi, G., Cole, S. T. & Manganelli, R. 2010 Development of a repressible mycobacterial promoter system based on two transcriptional repressors. *Nucleic Acids Res.* **38**, 1–11. (doi:10.1093/nar/gkq235)

2. Consaul, S. A. & Pavelka Jr, M. S. 2004 Use of a novel allele of the *Escherichia coli aacC4* aminoglycoside resistance gene as a genetic marker in mycobacteria. *FEMS Microbiol. Lett.* **234**, 297–301. (doi:10.1016/j.femsle.2004.03.041)

3. Pelicic, V., Jackson, M., Reyrat, J. M., Jacobs Jr., W. R., Gicquel, B. & Guilhot, C. 1997 Efficient allelic exchange and transposon mutagenesis in *Mycobacterium tuberculosis*. *Proc.Natl.Acad.Sci.U.S.A* **94**, 10955–10960.

4. Hanahan, D. 1983 Studies on transformation of *Escherichia coli* with plasmids. *J.Mol.Biol.* **166**, 557–580.

5. Snapper, S. B., Melton, R. E., Mustafa, S., Kieser, T. & Jacobs Jr., W. R. 1990 Isolation and characterization of efficient plasmid transformation mutants of *Mycobacterium smegmatis*. *Mol.Microbiol.* **4**, 1911–1919.

**Table S 3. RT primers used in this study.**

| **Oligonucleotide** | **Sequence** |
| --- | --- |
| **L-sigA** | ccaagggctacaagttctcg |
| **R-sigA** | tggatctccagcaccttctc |
| **L-fas** | cgatgcagaccatgtaccac |
| **R-fas** | cccacatacgactgcatgac |
| **L-acpS** | gtcgagctctcggttctcc |
| **R-acpS** | gtcacgtcctccaggtgttt |
| **L-fasR** | aggcgttcttcgatttcatc |
| **R-fasR** | atcagatcgaacacggcatc |
| **L-fabD** | cacacccactacatggcatc |
| **R-fabD** | gagttgtgcgacgagcttct |
| **L-acpM** | catcaagtccccagaagacc |
| **R-acpM** | tcgataccggtgacctcttc |
| **L-kasA** | aacccgaacgagttgttgat |
| **R-kasA** | ccgacgctgaactacgagac |
| **L-kasB** | aacaacctggatccggaaat |
| **R-kasB** | acattgtgcccaccgaat |
| **L-fabH** | gaccaggcacaacacacata |
| **R-fabH** | actgcaattgatcaccacct |
| **L-hadB** | cggagatctggtcaactacg |
| **R-hadB** | tgaaccgcacgttgtactc |
| **L-inhA** | gaggagcacctgtcgactct |
| **R-inhA** | acggtcatccagttgtaggc |
