# Supplementary material for: A conditional mutant of the fatty acid synthase unveils unexpected cross talks in mycobacterial lipid metabolism
Source: Open Biol. 2017 Feb 22;7(2):160277. doi: 10.1098/rsob.160277 (PMC5356441; doi:10.1098/rsob.160277)
Supplement: Figuras S1-S8 [file rsob160277supp3.pdf]

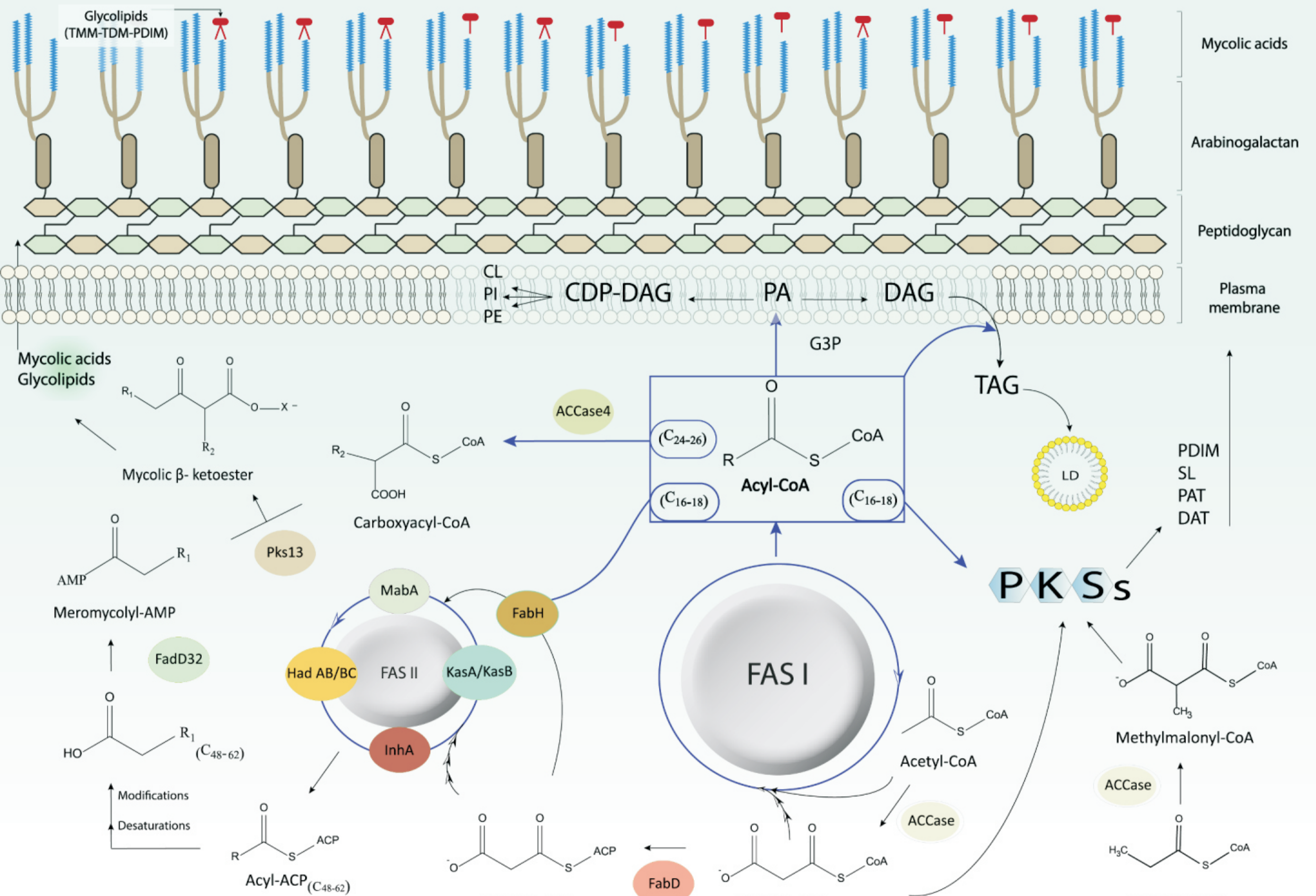

Glycolipids (TMM-TDM-PDIM)
Mycolic acids
Arabinogalactan
Peptidoglycan
Plasma membrane
CL
PI
PE
CDP-DAG
PA
DAG
G3P
TAG
LD
Mycolic acids
Glycolipids
Mycolic β- ketoester
ACCase4
Acyl-CoA
CoA
(C24-26)
(C16-18)
(C16-18)
Carboxyacyl-CoA
COOH
Pks13
Meromycolyl-AMP
AMP
FadD32
MabA
FabH
Had AB/BC
FAS II
KasA/KasB
InhA
FAS I
PKSs
PDIM
SL
PAT
DAT
Methylmalonyl-CoA
Acetyl-CoA
ACCase
ACCase
(C48-62)
Modifications
Desaturations
Acyl-ACP(C48-62)
Malonyl-ACP
FabD
Malonyl-CoA
Propionyl-CoA

Figure S1

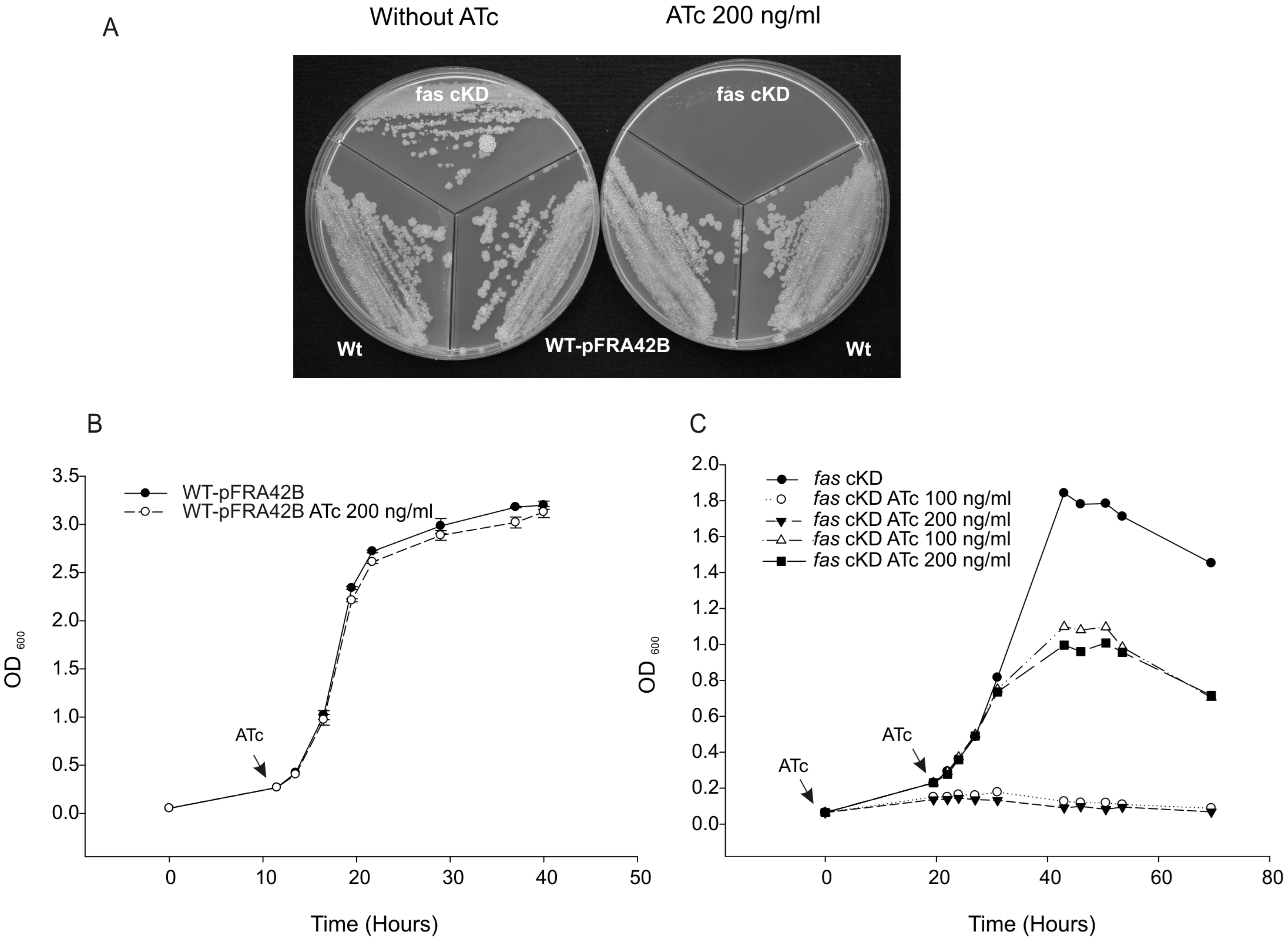
A
Without ATc
ATc 200 ng/ml
fas cKD
fas cKD
Wt
WT-pFRA42B
Wt
B
WT-pFRA42B
WT-pFRA42B ATc 200 ng/ml
ATc
OD 600
Time (Hours)
0.0
0.5
1.0
1.5
2.0
2.5
3.0
3.5
0
10
20
30
40
50
C
fas cKD
fas cKD ATc 100 ng/ml
fas cKD ATc 200 ng/ml
fas cKD ATc 100 ng/ml
fas cKD ATc 200 ng/ml
ATc
ATc
OD 600
Time (Hours)
0.0
0.2
0.4
0.6
0.8
1.0
1.2
1.4
1.6
1.8
2.0
0
20
40
60
80

Figure S2

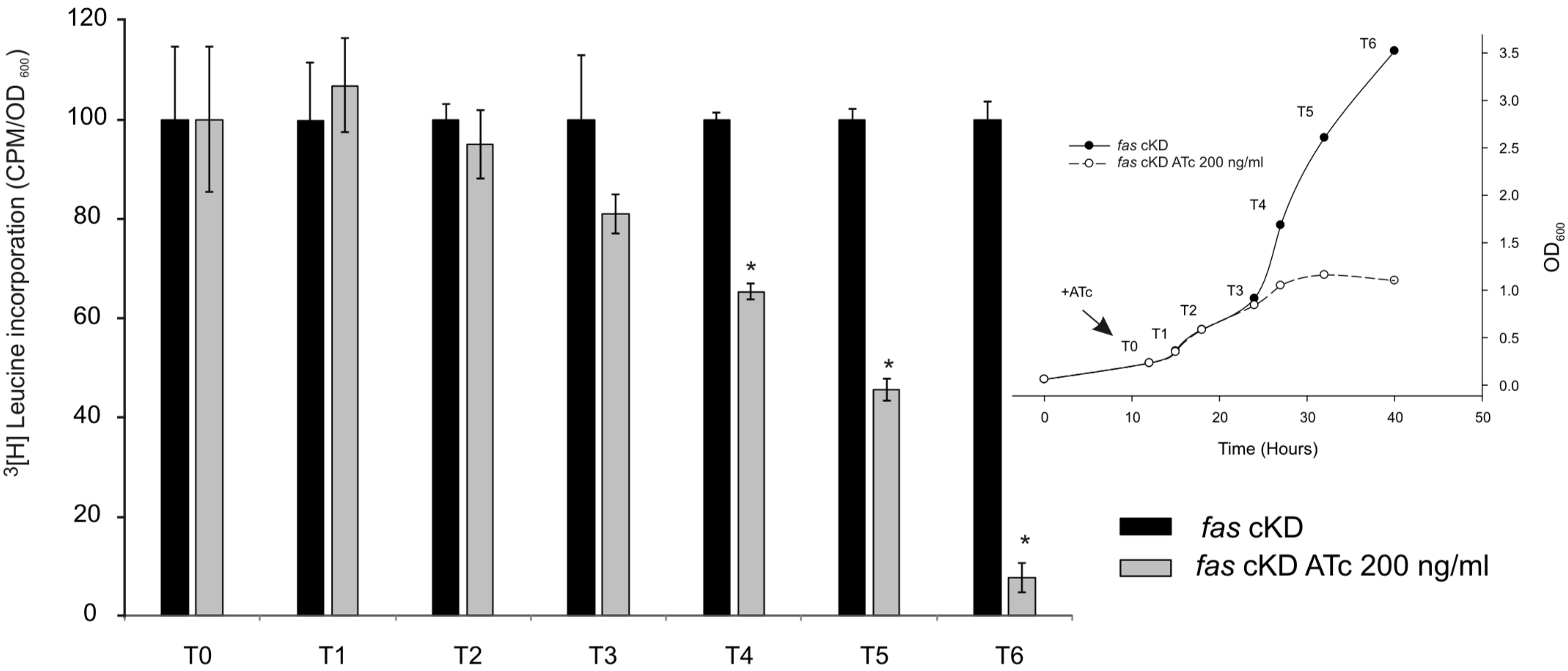

120
100
80
60
40
20
0
^{3}[H] Leucine incorporation (CPM/OD $_{600}$)
T0
T1
T2
T3
T4
T5
T6
*
fas cKD
fas cKD ATc 200 ng/ml
+ATc
0.0
0.5
1.0
1.5
2.0
2.5
3.0
3.5
OD_{600}
0
10
20
30
40
50
Time (Hours)
fas cKD
fas cKD ATc 200 ng/ml

Figure S3

Strain	% of:									
	C14	C16:1	C16	C17	C18:1	C18	C19	C20	C22	C24
fas cKD T3	0.9	6.12	32.65	0	16.74	0.99	15.56	0.25	1.74	24.41
fas cKD ATc 200 ng/ml T3	0.76	7.46	22.74	0	10.15	2.29	17.91	0	1.72	36.47
fas cKD T4	0.73	4.48	33.9	0.51	17	1.1	16.88	0.16	1.43	23.81
fas cKD ATc 200 ng/ml T4	0.61	7.71	22.68	0.51	10.31	2.88	17.68	0	1.42	36.05
WT-pFRA42B	1.03	5.11	32.52	0.37	22.25	1.11	13.67	0.38	2.47	21.04
WT-pFRA42B ATc 200 ng/ml	1.11	6.51	31.29	0.4	21.57	1.56	13.91	0.45	2.51	20.69

Figure S4

A

Weighted average	WT-pFRA42B ATc 200ng/ml	WT-pFRA42B	*fas* cKD ATc 200ng/ml	*fas* cKD	P value
MAα number of carbons	77.01 +/- 0.4	77.32 +/- 0.2	78.06 +/- 0.03	77.09 +/- 0.2	0.0163

B

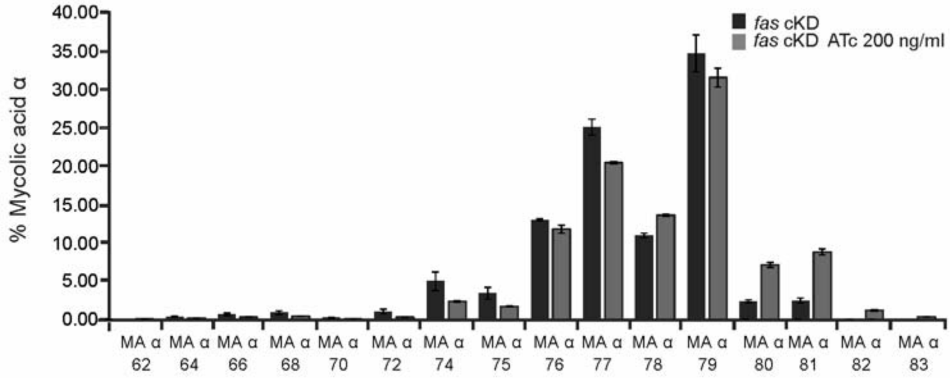

C

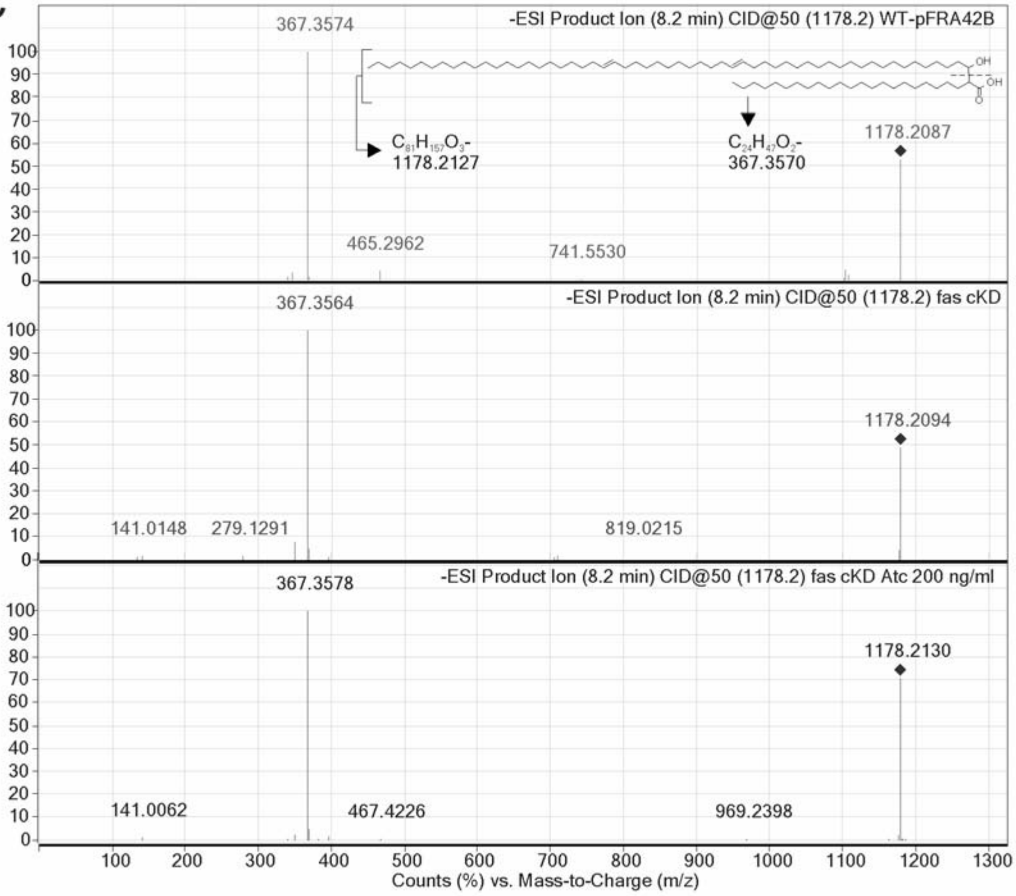

Figure S5

A

Weighted average	WT-pFRA42B ATc 200ng/mL	WT-pFRA42B	*fas* cKD ATc 200ng/mL	*fas* cKD	P value
TMM α number of carbons	77.54 +/- 0.2	77.65 +/- 0.04	78.59 +/- 0.04	77.54 +/- 0.04	<0.0001
GroMM α number of carbons	77.47 +/- 0.1	77.54 +/- 0.06	78.35 +/- 0.01	77.46 +/- 0.02	0.0019

B

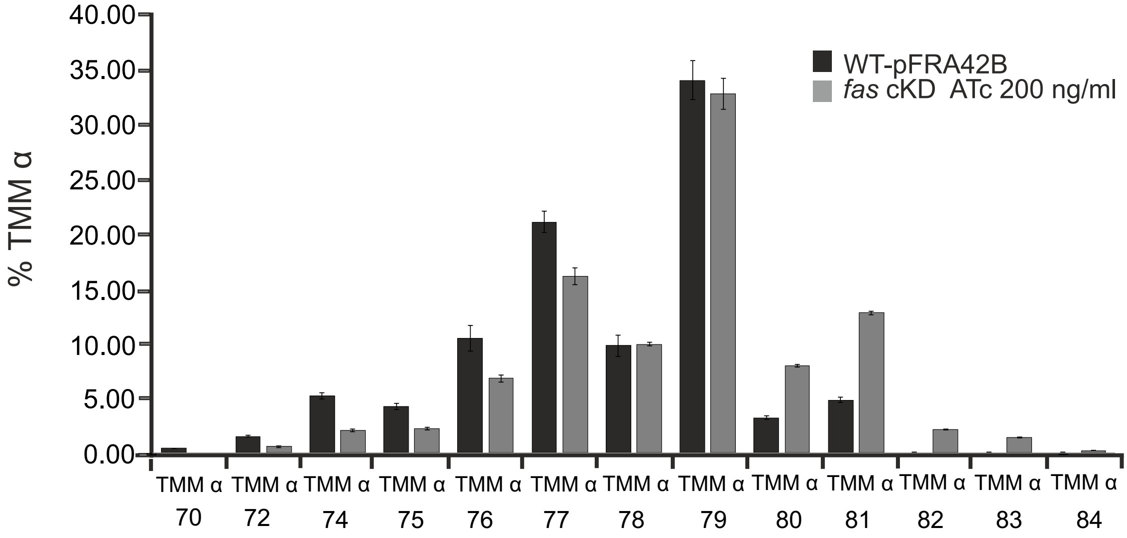

C

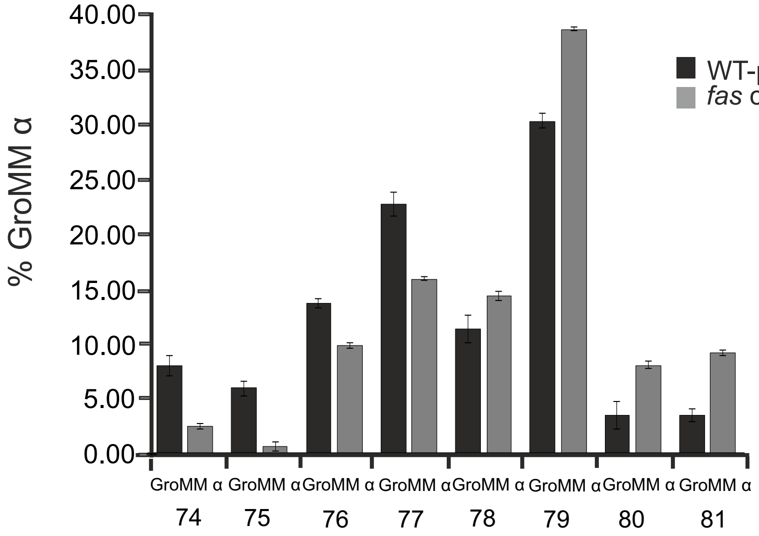

Figure S6

A

Weighted average	WT-pFRA42B ATc 200ng/mL	WT-pFRA42B	*fas* cKD ATc 200ng/mL	*fas* cKD	P value
**CL**	67.73 +/- 0.14	67.65 +/- 0.03	68.35 +/- 0.02	67.91 +/- 0.04	< 0.0001
**PE**	34.22 +/- 0.09	34.14 +/- 0.06	34.65 +/- 0.01	34.19 +/- 0.23	0.0034
**PG**	34.29 +/- 0.11	34.17 +/- 0.06	34.25 +/- 0.07	34.21 +/- 0.05	0.2035
**PI**	34.70 +/- 0.06	34.66 +/- 0.01	34.76 +/- 0.04	34.83 +/- 0.02	0.0523

B

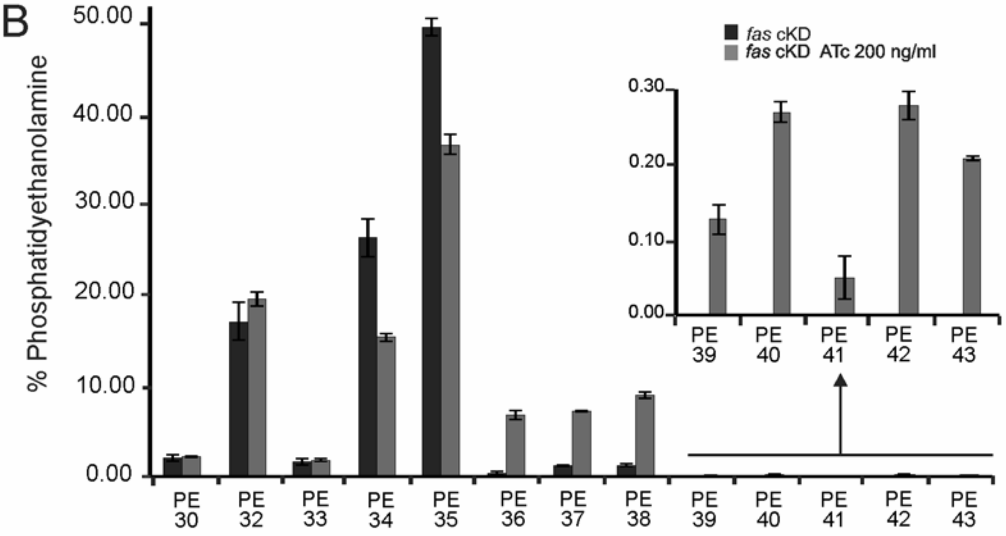

C

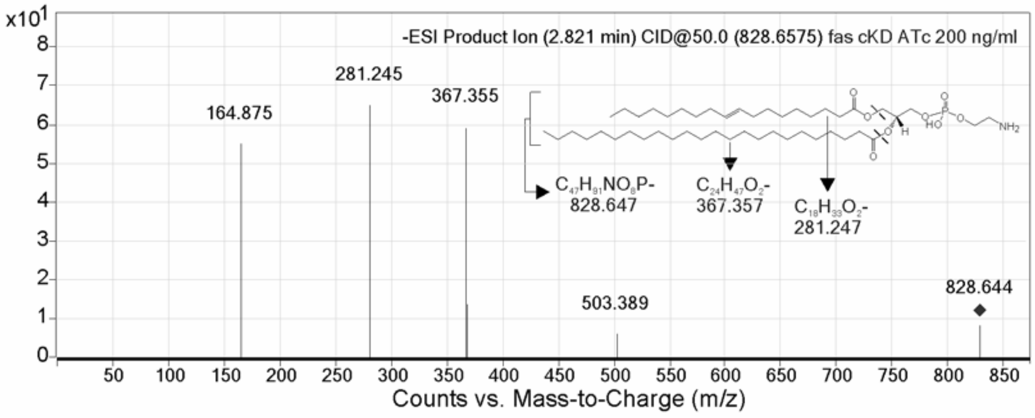

Figure S7

Cardiolipin

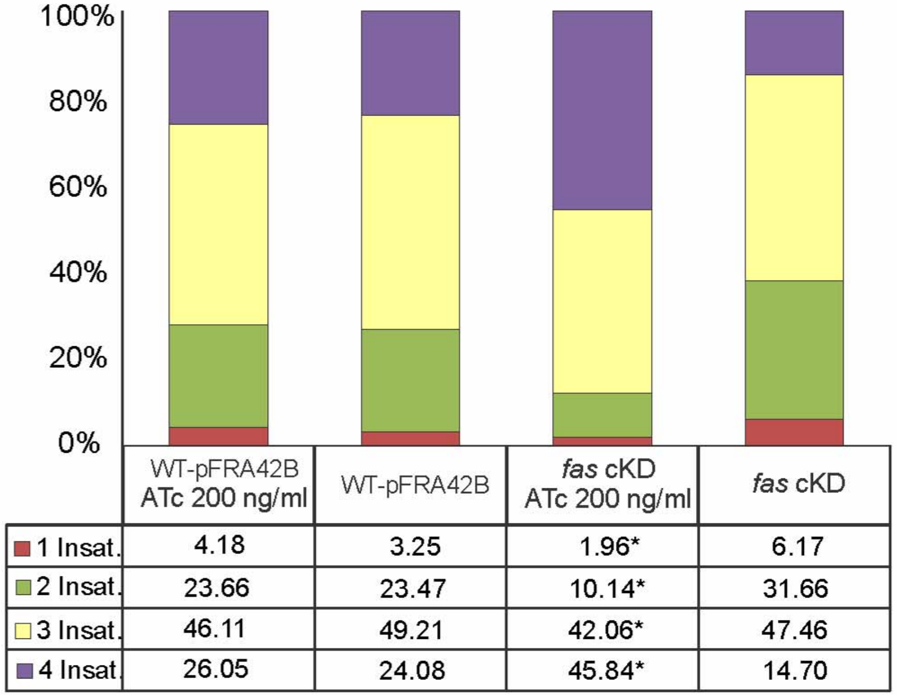

	WT-pFRA42B ATc 200 ng/ml	WT-pFRA42B	*fas* cKD ATc 200 ng/ml	*fas* cKD
1 Insat.	4.18	3.25	1.96*	6.17
2 Insat.	23.66	23.47	10.14*	31.66
3 Insat.	46.11	49.21	42.06*	47.46
4 Insat.	26.05	24.08	45.84*	14.70

Phosphatidylinositol

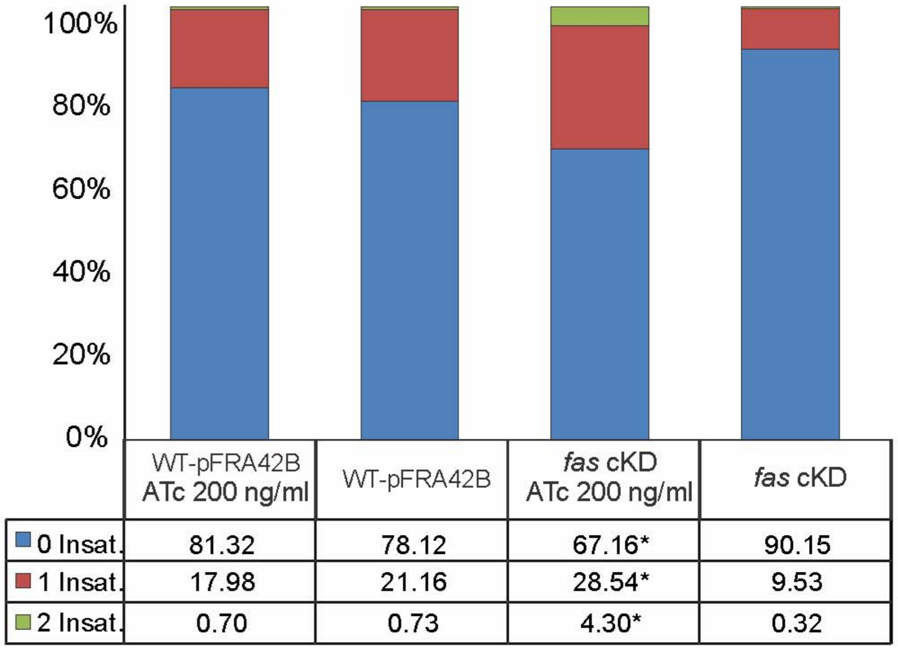

	WT-pFRA42B ATc 200 ng/ml	WT-pFRA42B	*fas* cKD ATc 200 ng/ml	*fas* cKD
0 Insat.	81.32	78.12	67.16*	90.15
1 Insat.	17.98	21.16	28.54*	9.53
2 Insat.	0.70	0.73	4.30*	0.32

Phosphatidylethanolamine

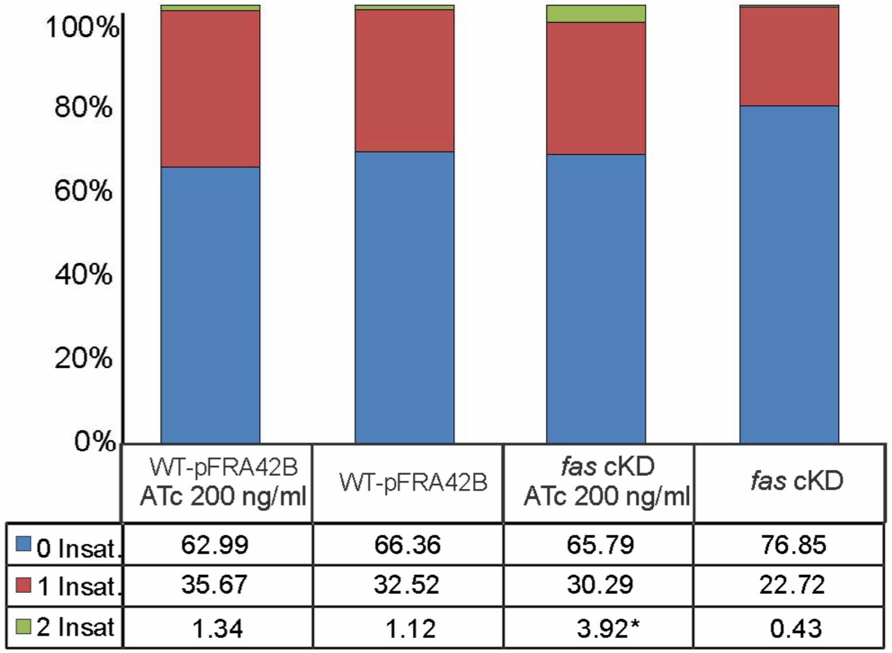

	WT-pFRA42B ATc 200 ng/ml	WT-pFRA42B	*fas* cKD ATc 200 ng/ml	*fas* cKD
0 Insat.	62.99	66.36	65.79	76.85
1 Insat.	35.67	32.52	30.29	22.72
2 Insat	1.34	1.12	3.92*	0.43

Phosphatidyl glycerol

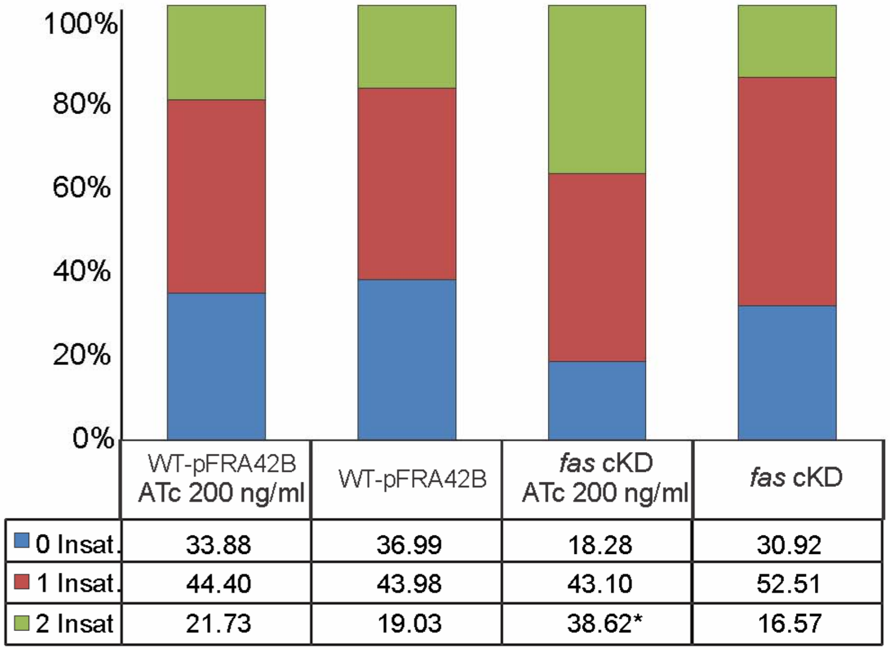

	WT-pFRA42B ATc 200 ng/ml	WT-pFRA42B	*fas* cKD ATc 200 ng/ml	*fas* cKD
0 Insat.	33.88	36.99	18.28	30.92
1 Insat.	44.40	43.98	43.10	52.51
2 Insat	21.73	19.03	38.62*	16.57

Figure S8
